# HCoV-229E does not trigger early interferon gene expression and evades IFN signaling in human A549 lung epithelial cells

**DOI:** 10.1128/spectrum.00510-25

**Published:** 2025-10-28

**Authors:** Rickard Lundberg, Anna Jartti, Jemna Heroum, Laura Kakkola, Ilkka Julkunen

**Affiliations:** 1 Institute of Biomedicine, University of Turku 8058, Turku, Finland; 2 Clinical Microbiology, Turku University Hospital 1041, Turku, Finland; 3 InFlames Research Flagship Center, University of Turku 8058, Turku, Finland; National University of Singapore, Singapore, Singapore

**Keywords:** coronavirus, innate immunity, virus-host interactions, immune evasion

## Abstract

Coronaviruses cause upper and lower respiratory tract infections ranging from mild infections to severe conditions such as pneumonia and acute respiratory distress syndrome (ARDS). One factor contributing to the pathogenesis of viral infection is the ability to avoid or downregulate the activation of interferon (IFN)-induced antiviral immunity. We show that the seasonal human coronavirus HCoV-229E readily replicated in human lung A549 and human hepatoma Huh7 cells, but the infection did not induce type I or type III IFN gene expression and IFN-induced MxA expression. Total cellular RNA isolated from HCoV-229E-infected A549 or Huh7 cells transfected into uninfected A549 cells failed to induce significant IFN gene expression, indicating that HCoV-229E RNA is likely not recognized by RIG-I-like receptors. However, in similar experiments, influenza A virus (IAV) infection or cellular RNA from IAV-infected cells induced strong IFN responses. Pretreatment of A549 cells with IFN-α showed some inhibitory activity against HCoV-229E replication, but the level of inhibition was weaker than that observed against IAV. Additionally, the ability of type I IFNs to induce MxA protein expression was almost completely blocked in HCoV-229E-infected A549 cells. This phenomenon was observed both at early (8 h) and late (24 h) time points of HCoV-229E infection, indicating that the infection efficiently blocks IFN signaling. Our data show that HCoV-229E can efficiently avoid cellular IFN responses.

**IMPORTANCE:**

This study uncovers some of the potential mechanisms by which the seasonal coronavirus HCoV-229E evades host innate immune responses, providing valuable insights into general coronavirus pathogenesis. Unlike IAV, HCoV-229E infection does not induce type I IFN production and IFN-induced antiviral MxA expression. Cellular RNA from HCoV-229E-infected cells fails to induce IFN gene expression, indicating that HCoV-229E virus-specific RNA molecules are not recognized by pattern recognition receptors (PRRs). This immune evasion allows the virus to replicate and produce proteins that further inhibit host defenses. These findings challenge prior reports on IFN responses to seasonal coronaviruses and highlight differences in immune evasion between seasonal and more pathogenic coronaviruses like SARS-CoV-2. By elucidating these mechanisms, the study paves the way for identifying antiviral targets across the coronavirus family and understanding the balance between immune evasion and disease severity, ultimately contributing to the development of broad-spectrum antiviral strategies.

## INTRODUCTION

Coronaviruses (CoVs) are a diverse family of viruses that infect a wide variety of animals. The family consists of four genera: *alpha-, beta-, gamma-,* and *deltacoronaviruses*, of which alpha- and betacoronaviruses infect mammals. There are seven coronaviruses known to infect humans: severe acute respiratory syndrome coronavirus (SARS-CoV), SARS-CoV-2, Middle East respiratory syndrome coronavirus (MERS-CoV), and human coronaviruses HCoV-229E, -HKU1, -NL63, and -OC43. From these seven, SARS-CoV, SARS-CoV-2, and MERS-CoV have emerged since 2002 and are highly or moderately (SARS-CoV-2) pathogenic to humans, whereas the alphacoronaviruses HCoV-229E and HCoV-NL63 and betacoronaviruses HCoV-OC43 and HCoV-HKU1 have circulated globally for a longer time and are usually associated with mild upper respiratory tract infections. However, they can result in more severe symptoms, such as pneumonia or meningoencephalitis, especially in immunosuppressed patients, infants, and the elderly (1, 2).

The species HCoV-229E was isolated in 1965 (3). The virus likely emerged from a bat coronavirus, potentially having camelids as an intermediate host species before becoming established as a human pathogen (4). Coronaviruses are enveloped, single-stranded, nonsegmented, and positive-sense RNA viruses with genomes ranging from 27 to 32 kilobases, making them the largest known RNA viruses infecting humans. Coronaviruses have a highly conserved genomic organization with a 5′ cap structure and a 3′ poly(A) tail (5, 6).

In most cases, viral infection activates the innate immune response in infected cells. There are three classes of interferons: type I, II, and III IFNs, where type I (IFN-α, -β) and type III (IFNs λ1-3/4) IFN gene expressions are activated in response to virus infection. The invading virus is recognized by PRRs such as RIG-I and Toll-like receptors that are activated by viral nucleic acids or proteins. Interferons are secreted by virus-infected cells and bind to IFN receptors on the surface of the same and neighboring cells. This leads to the activation of Janus kinase (JAK) and signal transducer and activator of transcription (STAT) pathway, where transcription factors STAT1 and STAT2 together with interferon regulatory factor (IRF) 9 form the interferon-stimulated gene factor (ISGF) 3 complex, which translocates into the nucleus and binds to promoter sequences called interferon signaling response elements (ISRE). This results in the expression of genes collectively known as interferon-stimulated genes (ISGs) (7).

Myxovirus resistance (Mx) proteins are dynamin-like GTPases that play key roles in the innate immune response by interfering with the early stages of the viral replication cycle. The expression of MxA, one of the two principal Mx genes in humans, is induced and regulated by type I (IFN-α/β) and type III (IFN-λ) IFNs (8–11). Importantly, since Mx proteins are not constitutively expressed and are not induced directly by virus factors, they are good markers for IFN action induced by, for example, virus infection. MxA is a central mediator of IFN-induced antiviral response against a broad range of viruses, interfering with different viruses by different mechanisms. For example, in the case of bunyaviruses, MxA inhibits the virus life cycle by sequestering nascent viral N proteins into perinuclear complexes, and while it is in the case of IAV not fully understood, it is proposed that MxA prevents the viral nucleoprotein complexes from entering the nucleus and/or by preventing transcription of viral genomic RNA to mRNA in the nucleus (12–15).

At present, the interaction between seasonal coronaviruses and the host IFN system is poorly characterized. In this study, we analyzed the early (2–24 hpi) innate immune responses in A549 and Huh7 cells following infection with the seasonal coronavirus HCoV-229E to provide key insights into immune evasion strategies employed by seasonal coronaviruses. Additionally, we chose to include IAV as a control due to its well-characterized and robust IFN induction in A549, while HCoV-229E was studied to elucidate seasonal coronavirus immune evasion, distinct from highly pathogenic coronaviruses like SARS-CoV-2, which require different cell models. We showed that, unlike IAV, HCoV-229E infection does not induce the expression of IFNs and IFN-induced MxA. Additionally, IFN-α-induced MxA protein expression was dramatically reduced in HCoV-229E virus-infected cells.

## MATERIALS AND METHODS

### Cell cultures

A549 human lung carcinoma cells (ATCC CCL-185) and hepatocellular carcinoma Huh7 cells (16) were maintained in Dulbecco’s modified Eagle’s medium (DMEM; EuroClone) supplemented with penicillin/streptomycin (EuroClone), L-glutamine (Gibco), and 10% heat-inactivated fetal bovine serum (Gibco). All cells were maintained at 37°C in a humidified atmosphere in the presence of 5% CO_2_.

### Antibodies

HCoV-229E N protein (α-N) and IAV virus nucleoprotein (α-NP) specific rabbit antibodies used in immunoblots and immunofluorescence assays were as previously described (17, 18). The specificities of in-house produced rabbit and guinea pig α-MxA antibodies have been described previously (19, 20). Mouse monoclonal α-MxA (InVivo BioTech Services) was used according to the manufacturer’s instructions. Mouse α-GAPDH (6C5; Santa Cruz Biotechnology) was used as a loading control in all immunoblots according to the manufacturer’s instructions.

### Viruses, infections, transfections, and stimulations

HCoV-229E (GenBank accession number: OK662398) was grown in Huh7 cells at 33°C for 48 hours (Fig. S1). The virus titer was determined by a 50% tissue culture infectious dose (TCID_50_) end-point assay according to the Reed-Muench method and results were converted to theoretical pfu/mL by multiplying by 0.69 (21, 22). The stock TCID_50_ end-point titer was estimated to be 1.77 × 10^7^ pfu/mL. IAV (A/California/07/2009 H1N1; provided by the Finnish Institute for Health and Welfare, THL, Finland) virus was grown in chicken eggs for 3 days, and the virus titer in the collected allantois fluid was determined by plaque assay in MDCK cells. The stock titer was 4.8 × 10^7^pfu/mL.

A549 or Huh7 cells were infected with different doses of HCoV-229E or IAV, and cellular samples were collected at time points after infection, as indicated in the respective experiments. Cells were processed for immunoblotting, immunofluorescence, or RNA analyses, as described below.

On a 12-well format, 80% confluent A549 cells were transfected with total cellular RNA isolated from uninfected and virus-infected cells (100 ng/mL) with Lipofectamine 2000 (Invitrogen) according to the manufacturer’s instructions using OptiMem media (Gibco). At time points 2, 4, 8, and 24 hours post-transfection, the cells were washed once with phosphate-buffered saline (PBS; Cytiva) and collected into RLT buffer (Qiagen), and total cellular RNA was isolated.

To stimulate the IFN-induced pathway, PEGylated interferon alpha-2a (IFN-α2a; Pegasys, Hoffman-La Roche) was added to the cell culture medium at indicated concentrations. To analyze the sensitivity of A549 and Huh7 cells to IFNs, the cells were stimulated with different doses of IFN-α2a (1-100 ng/mL) followed by collection of cells after 24 h stimulation and processing the samples for immunoblotting for IFN-induced MxA protein expression (see below; Fig. S2).

### Immunofluorescence assays

To study the kinetics of virus infection and protein expression, A549 and Huh7 cells were grown on 96- (without coverslips) and 12- or 6-well (with coverslips) format and treated as indicated in the figure legends. Following treatment, cells were washed once with PBS and fixed in 4% paraformaldehyde in PBS for 30 minutes at room temperature. The fixed cells were blocked and permeabilized in 0.5% bovine serum albumin (BSA; Sigma-Aldrich) and 0.1% Triton X-100 (VWR International) in PBS for 30 minutes at room temperature. The blocking/permeabilization solution was removed, and primary antibodies (in-house rabbit α-IAV-NP in 1:1,000 or α-HCoV-229E-N in 1:2,500, or guinea pig α-MxA or monoclonal mouse α-MxA in 1:1,000 or 1:16,000 dilution, respectively) in 3% BSA in PBS were added followed by an incubation for 1 hour at room temperature. The cells were washed thrice for 10 minutes in PBS. Secondary antibodies [goat α-rabbit IgG (H + L) AlexaFluor Plus 488, goat α-mouse IgG (H + L) AlexaFluor Plus 568, or goat α-guinea pig IgG (H + L) AlexaFluor Plus 568, Thermo Fisher Scientific] and 4′,6-diamidino-2-phenylindole (DAPI; Life Technologies) in 3% BSA in PBS were added followed by an incubation for 1 hour at room temperature in the dark. The cells were washed thrice for 10 minutes in 3% BSA in PBS and once with only PBS. In the case of the 96-well format, the cells were imaged using the EVOS FL Auto imaging system (Life Technologies). In case of the 12- and 6-well formats, the coverslips were mounted onto glass slides using ProLong Gold Antifade Mountant (Invitrogen) and visualized with Leica DMLB 100 s microscope with a DFC7000 T camera and/or Zeiss LSM880 confocal microscope. The images were processed and quantified in Fiji, which is an open-source distribution of ImageJ (23). To obtain estimates of infection percentages, the infection was quantified by training a binary classifier (Trainable Weka Segmentation plugin in Fiji [24]), and the nuclei of virus-infected cells were compared to the total number of nuclei for each image, which were segmented with the StarDist2D plugin in Fiji (25). For training of the algorithm, 20% of the images from the first repetition were used, and the remaining 80% from the same repetition were used for validation. Where applicable, the resulting classifier was used to estimate the percentages of infection in images taken at random locations on slides or in wells from subsequent repetitions.

### Immunoblotting

For protein analyses, whole-cell lysates from infected, transfected, or stimulated cells on 6-well format were collected into Passive Lysis Buffer provided in the Dual Luciferase Assay Kit (Promega) supplemented with Pierce protease inhibitors (Thermo Fisher Scientific) and Pierce Universal Nuclease for cell lysis (Thermo Fisher Scientific). The total protein concentration was measured using a Bradford assay kit (BioRad), and equal amounts (20 or 30 µg per lane) of protein were loaded onto either precast Mini-Protean TGX any kD (BioRad) or fresh cast SDS-PAGE gels. Following SDS-PAGE protein separation at 150 V in commercial SDS running buffer (BioRad), Amersham Hybond-P 0.2 µm polyvinylidene difluoride (PVDF) membranes (Cytiva) were activated in 100% methanol, and the proteins from SDS-PAGE gels were transferred onto PVDF membranes by tank transfer at constant 20 V for 20 hours. The membranes were blocked in 5% BSA in Tris-buffered saline (TBS). Immunoblotting was done according to the manufacturer’s instructions for a particular antibody. Secondary antibodies were IRDye 800CW goat α-rabbit IgG and IRDye 680RD goat α-mouse IgG (LI-COR Biosciences). Membranes were scanned with Odyssey Fc Imaging System (LI-COR Biosciences). The resulting images were processed in Fiji.

### Quantitative reverse-transcription polymerase chain reaction (RT-qPCR)

Cells infected with the indicated virus or transfected with indicated RNA samples were harvested using RLT buffer (Qiagen), and total cellular RNA was isolated using the RNEasy kit (Qiagen). Total cellular RNA (500 ng) was transcribed into cDNA using the RevertAid H Minus First Strand cDNA Synthesis Kit according to the manufacturer’s instructions (Thermo Fisher Scientific). The resulting cDNAs were amplified by PCR using the TaqMan Universal PCR Mastermix along with appropriate gene expression assays (CXCL10, Hs00171042_m1; IFN-β, Hs00277188_s1; IFN-λ, Hs00601677_g1; GAPDH, Hs02758991_g1; hMxA, Hs00895608_m1; IFN-α1, Hs00256882_s1; IFN-α2, Hs00265051_s1; Applied Biosystems). For the detection of IAV mRNA levels, the following IAV virus-specific M1 primer pair/probe setup was used: forward primer 5′-TTG AGG CTC TCA TGG AAT GG, reverse primer 5′-CAA AGC GTC TAC GCT GCA GTC, probe FAM-5′-TTT GTG TTC ACG CTC ACC GT-BHQ1; for HCoV-229E, the following HCoV-229E-specific N primer pair/probe setup was used: forward primer 5′-CGT AAT CAG AGT CCT GCT TC, reverse primer 5′-CAC AAC ACC TGC ACT TCC, probe FAM-5′-TCA AAC TTC TGC CAA GAG TCT TGC TCG T-BHQ1. The results are presented as relative gene expression units using the ddC_t_ method. Compared to mock cells, or 2 hours post-infection cells for the viruses, the C_t_ values were normalized to GAPDH levels, resulting in a dC_t_ value (i.e., dC_t_ = C_tSample_ – C_tGAPDH_). A ddC_t_ value is then calculated (ddC_t_ = dC_tSample_ – dC_tMock_), and the results are expressed as fold changes compared to the mock samples (2^-ddCt^).

### Statistics

The χ²-tests that were used to assess the significance of the findings in Fig. 5 and 6 were done in GraphPad Prism (version 10.0.0 for Windows, GraphPad Software, Boston, MA, USA, https://www.graphpad.com/).

## RESULTS

### HCoV-229E infection in A549 and Huh7 cells does not induce the expression of antiviral MxA protein

**Fig 1:**
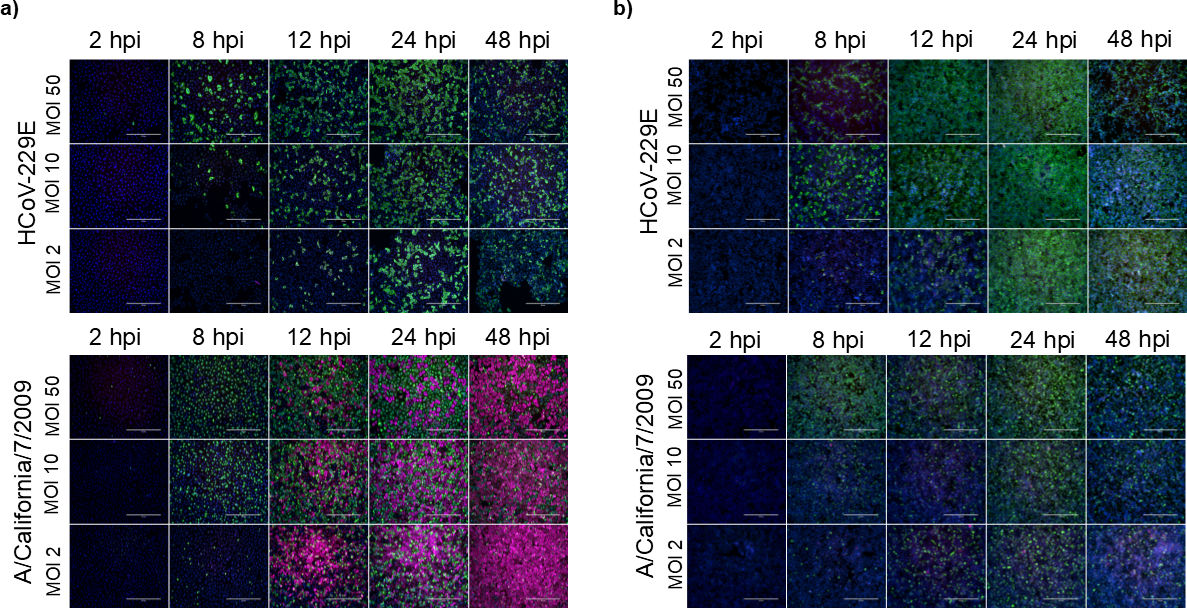
Infection kinetics and induction of MxA expression in A549 and Huh7 cells infected with HCoV-229E or influenza A virus. (**a**) A549 or (**b**) Huh7 cells were infected with HCoV-229E (top) or IAV (bottom) viruses using different MOI values, as indicated in the figure (MOI 50, 10, or 2). The cells were fixed at indicated time points. Virus infection was detected with rabbit α-229E-N or α-IAV-NP antibodies (green), and MxA was detected with guinea pig α-MxA antibodies (magenta). The experiment was repeated three times with similar results. Viral and MxA protein expression patterns are from one individual experiment. Scale bar, 400 µm.

To assess the infection kinetics of HCoV-229E in A549 and Huh7 cells, both cell lines were infected with three multiplicities of infection (MOI 2, 10, and 50) of HCoV-229E. The influenza A virus (strain A/California/07/2009), which is known to readily infect both cell types (26), was used as a control with the same MOI values. The kinetics of infection were analyzed by immunofluorescence at time points ranging from 2 to 48 hours post-infection (Fig. 1). HCoV-229E and IAV readily infected both cell lines. While IAV replication was comparable in both cell lines, HCoV-229E replication was more robust in Huh7 cells compared to that in A549 cells (additional dilutions and time points available in Fig. S3).

To study whether HCoV-229E infection induces antiviral MxA protein expression, infected cells were imaged for viral and MxA protein expression (Fig. 1). Interestingly, HCoV-229E infection did not induce any detectable MxA expression in A549 cells, whereas IAV infection induced a significant and temporally patterned expression of MxA (Fig. 1a). With IAV, infection at MOI 2 induced the highest MxA expression at 12–48 hpi, possibly due to optimal IFN induction in uninfected cells, whereas higher MOIs accelerated infection, reducing MxA expression (27). MxA expression was not detected in Huh7 cells in response to infection with either virus (Fig. 1b).

**Fig 2:**
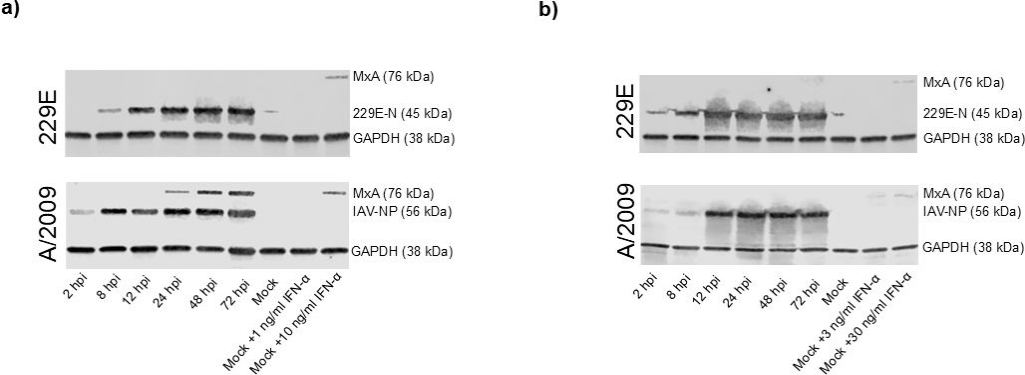
Cumulative production of virus-specific proteins and MxA in A549 and Huh7 cells infected with HCoV-229E or influenza A viruses. (**a**) A549 cells were infected with HCoV-229E (top, MOI 10) or IAV viruses (bottom, MOI 10). (**b**) Huh7 cells were infected with HCoV-229E (top, MOI 2) or IAV (bottom, MOI 10). Cells were collected at indicated time points, whole-cell lysates were prepared, and proteins were separated on SDS-PAGE, transferred to PVDF membranes, and virus proteins were then detected with rabbit α-229E-N and α-IAV-NP antibodies, and MxA was detected with guinea pig α-MxA antibodies. GAPDH was used as a loading control. The experiment was repeated three times with similar results. Representative protein expression patterns are shown from one experiment.

To validate the findings of the immunofluorescence assays, whole-cell lysates from the same time points were analyzed by immunoblotting (Fig. 2). As a control for MxA induction, A549 and Huh7 cells were treated with PEGylated IFN-α2a (0.1–100 ng/mL) for 24 hours. In A549 cells, the expression of MxA peaked between 3 and 10 ng/mL of IFN-α stimulation, and an increase in IFN-α concentrations did not further enhance MxA expression (Fig. S2). In Huh7 cells, instead, an IFN-α concentration of 30 ng/mL was required for detectable MxA expression, indicating that the antiviral response in Huh7 cells is dependent on higher amounts of IFN-α. In A549 cells, IAV infection resulted in time- and dose-dependent accumulation of virus-specific nucleoprotein (NP) and strong expression of MxA protein (Fig. 2a). In contrast, following HCoV-229E infection in A549 cells, while cumulative virus protein production was detectable from 8 hours post-infection onward, no detectable levels of MxA protein were observed. Similar to the immunofluorescence analysis, no detectable expression of MxA protein in Huh7 cells was observed by immunoblot following infection by either virus (Fig. 2b), despite both viruses demonstrating active replication in Huh7 cells.

### HCoV-229E infection in A549 and Huh7 cells does not induce cytokine gene expression

**Fig 3:**
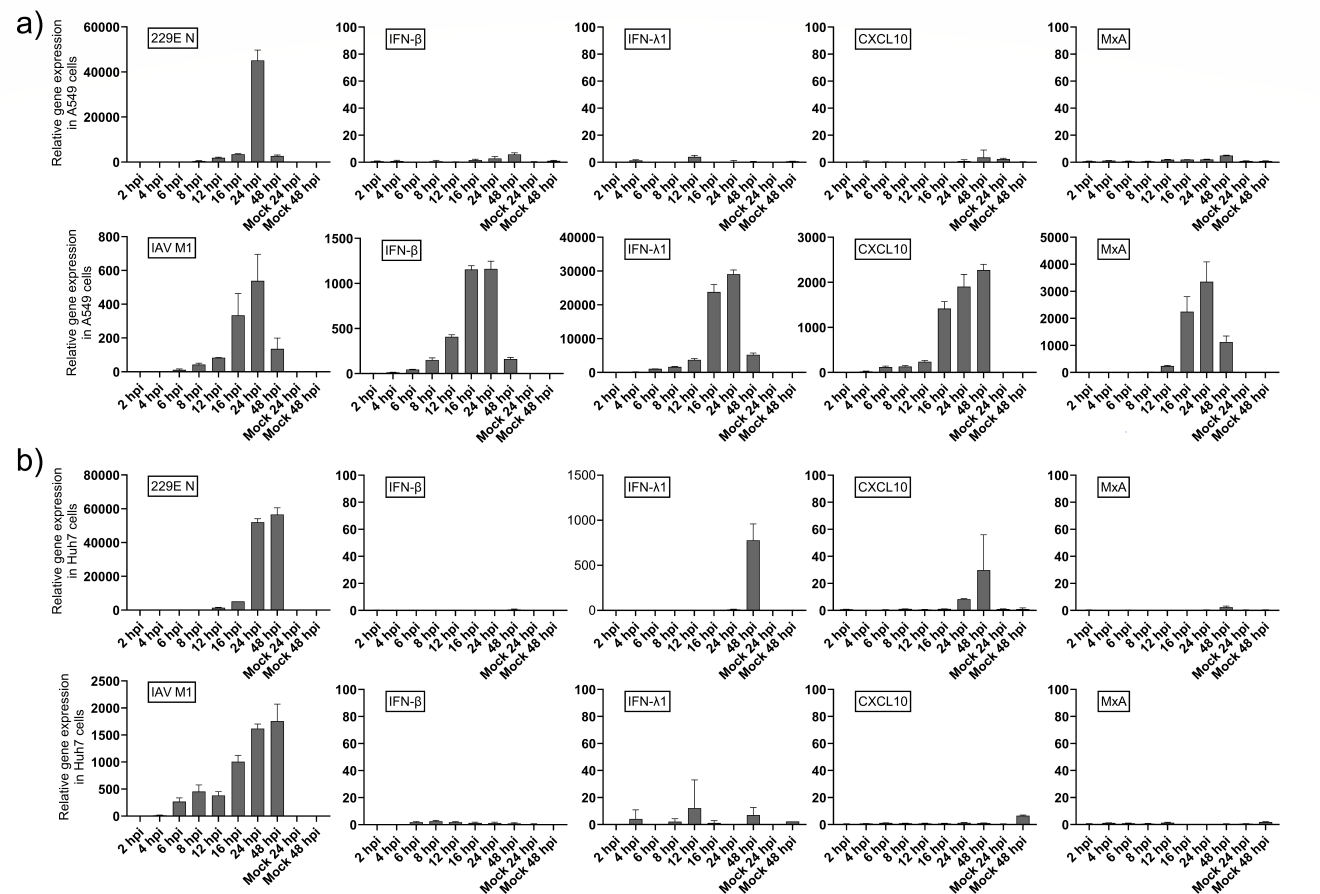
HCoV-229E infection in A549 or Huh7 cells does not induce the expression of cytokine genes. (**a**) A549 cells and (**b**) Huh7 cells were infected with HCoV-229E (top, MOI 10 for A549 and MOI 2 for Huh7) or IAV (bottom, MOI 10 for both A549 and Huh7) viruses. Cells were collected at different time points after infection, total cellular RNA was isolated, and the expression of viral RNAs and IFN-β, IFN-λ1, CXCL10, and MxA mRNA was analyzed with RT-qPCR. All the relative values were calibrated using the 2 hpi mock sample value or, in the case of virus-specific probes, the respective 2 hpi value as the calibrator. The error bars represent the standard deviation from the average of three technical replicates from one individual experiment. The experiment was repeated three times with similar results.

To further evaluate the potential of HCoV-229E infection to elicit cytokine gene expression, A549 and Huh7 cells were infected with HCoV-229E (MOI 50 in A549 cells; MOI 2 in Huh7 cells) and with IAV (MOI 10 for both A549 and Huh7 cells) as a control. The relative expression levels of IFN-β, IFN-λ1, CXCL10, MxA, and virus-specific N (for HCoV-229E) and M1 (for IAV) mRNAs were analyzed at indicated time points (2–48 hpi) (Fig. 3). In the case of IAV infection, there was a strong induction of IFN-β mRNA expression followed by induction of the MxA gene. IFN-λ1 and CXCL10 mRNAs were also readily induced (Fig. 3a, bottom). The lack of induction with mock RNA confirms that transfection alone does not trigger IFN gene expression, unlike synthetic immunogenic RNA such as poly(I:C). Interestingly, despite robust viral replication (detected by HCoV-229E N gene expression), there was practically no expression of cytokine or MxA genes in A549 cells following HCoV-229E infection (Fig. 3a, top). Moreover, despite active virus replication (detected with the expression of the HCoV-229E N or IAV M1 genes), neither virus induced any significant cytokine or MxA gene expression in Huh7 cells within 24 hours post-infection (Fig. 3b). However, in Huh7 cells infected with HCoV-229E, there was an increase in IFN-β and IFN-λ1 gene expressions at the late time point, 48 hours post-infection. This increase is most likely attributed to the heavy cytopathic effect caused by HCoV-229E infection in Huh7 cells from 48 hpi onward, and in keeping with the focus on early time points, RT-qPCR was restricted to 48 hpi due to extensive CPE in Huh7, making comparisons between cell lines and viruses difficult (Fig. 1b; Fig. S3). However, late IFN expression at 48 hpi may reflect CPE or delayed IFN signaling, as seen in SARS-CoV-2 (28), though our focus on early infection precludes stress sensor analysis.

### Total cellular RNA from HCoV-229E-infected cells does not induce IFN gene expression

**Fig 4:**
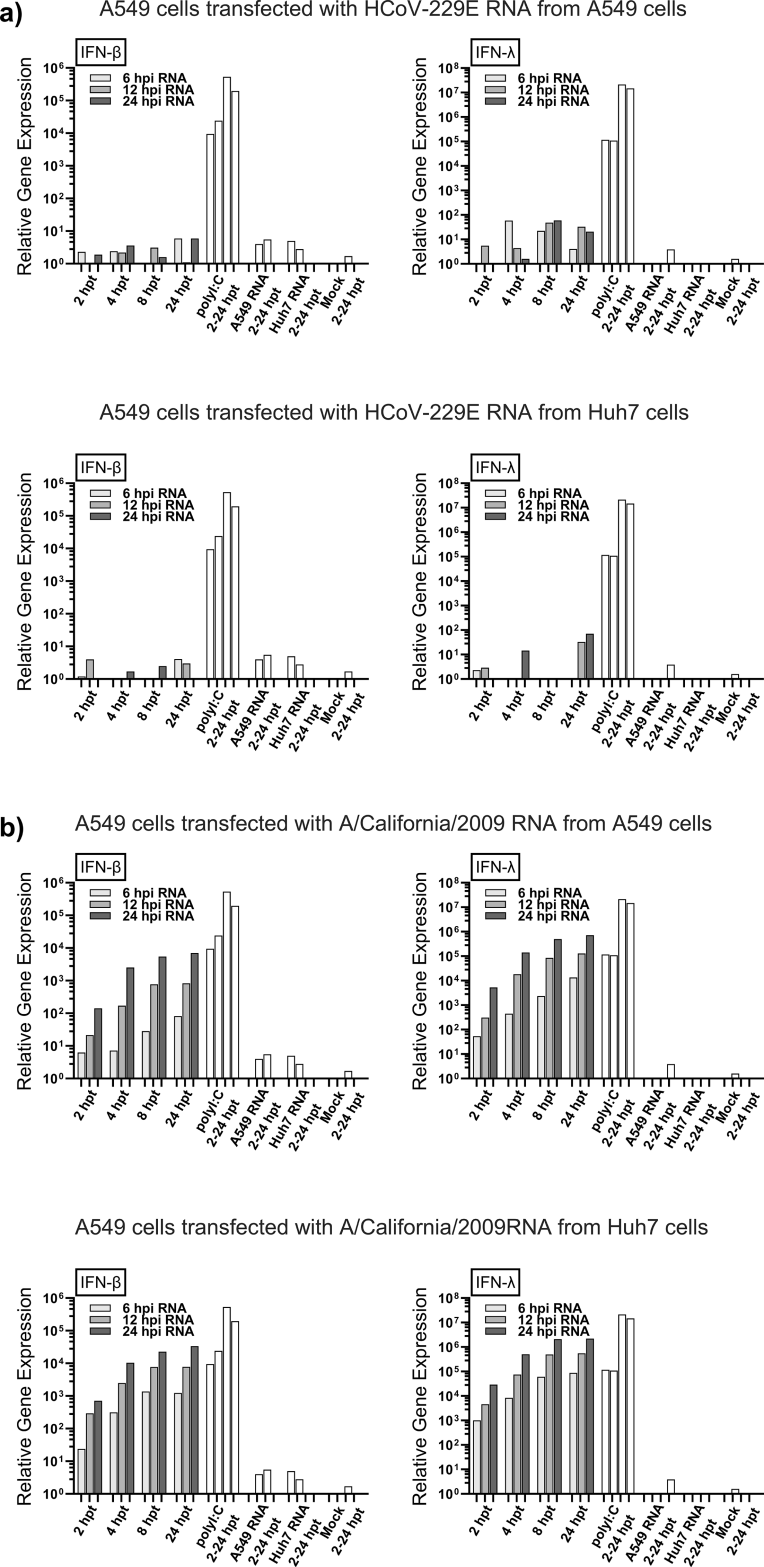
HCoV-229E RNA from infected A549 or Huh7 cells does not induce the expression of IFN genes in A549 cells. (**a**) Total cellular RNA collected at 6, 12, and 24 hpi from HCoV-229E-infected A549 cells (top, MOI 10) or Huh7 cells (bottom, MOI 2) was transfected (100 ng/well on a 12-well format) into A549 cells and collected at time points 2–24 hours post transfection (hpt). (**b**) Total cellular RNA collected at 6, 12, and 24 hpi from IAV-infected A549 cells (top, MOI 10) or Huh7 cells (bottom, MOI 10) was transfected (100 ng/well on a 12-well format) into A549 cells. Transfected cells were collected at different time points after transfection, total cellular RNA was isolated, and the expressions of IFN-β or IFN-λ1 genes in transfected A549 cells were analyzed by RT-qPCR for indicated time points. Poly(I:C) and total cellular RNA from uninfected cells (Mock) were included as controls. The relative values were calibrated using the 2 HPI A549 mock value. The experiment was repeated three times with similar results.

To investigate whether the viral RNA species produced during HCoV-229E infection can induce the IFN response, A549 and Huh7 cells were infected with HCoV-229E (MOI 50 for A549 cells and MOI 2 for Huh7 cells) or IAV as a positive control (MOI 10 for both A549 and Huh7 cells). Total cellular RNA was isolated from mock- and virus-infected cells collected at 6, 12, and 24 hpi, followed by transfection of equal amounts of total cellular RNA into A549 cells (100 ng/well on a 12-well format). To analyze whether the transfected total cellular RNA from infected cells was able to induce IFN gene expression in A549 cells, the expressions of IFN-β and IFN-λ1 mRNAs were analyzed by RT-qPCR (Fig. 4). As a positive control, A549 cells were transfected with poly(I:C), and as a negative control, cells were transfected with total cellular RNA from uninfected cells. As expected, poly(I:C) induced strong expression of both IFN-β and IFN-λ1 genes in A549 cells, whereas total cellular RNA from uninfected A549 or Huh7 cells did not induce significant IFN gene expression in A549 cells (Fig. 4). Total cellular RNA collected from A549 or Huh7 cells infected with IAV efficiently stimulated the expressions of IFN-β and IFN-λ1 genes in A549 cells (Fig. 4b). The induction of the expression of IFN genes was stronger with total cellular RNA isolated from the late-time-point infection samples. Interestingly, total cellular RNA from HCoV-229E-infected A549 or Huh7 cells did not induce IFN-β or IFN-λ1 gene expression in A549 cells (Fig. 4a). The expression of IFNs was not induced even with the total cellular RNA from the late time point of infection (24 hpi), when HCoV-229E replication in both cell lines is highly active (Fig. 1 to 3). These results strongly suggest that the viral RNA produced during HCoV-229E infection does not trigger IFN gene expression.

### Type I IFN pretreatment can weakly reduce HCoV-229E infection in A549 and Huh7 cells

**Fig 5:**
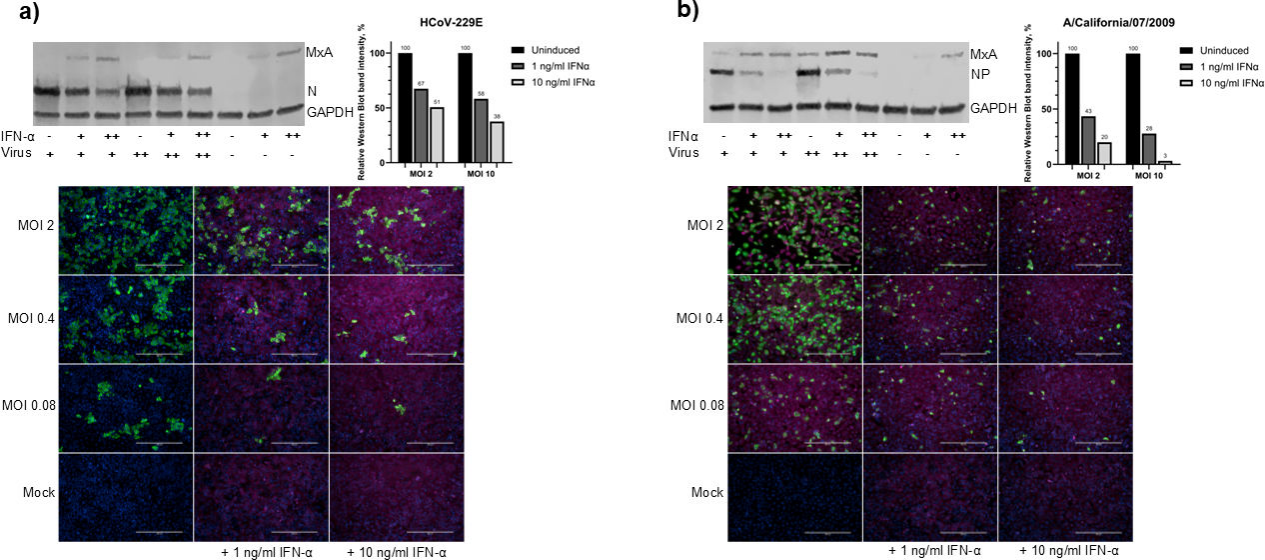
The effect of IFN-α pretreatment on HCoV-229E and IAV infection in A549 cells. (**a**) Immunoblot (top) of whole-cell lysates collected from A549 cells stimulated for 24 hours with 0, 1, or 10 ng/ml of IFN-α followed by infection with HCoV-229E (MOIs 2 and 10). With increasing IFN-α doses, enhanced expression of MxA (76 kDa) is seen. Pretreatment of cells with 1 or 10 ng/mL of IFN-α leads to a significant reduction in HCoV-229E N (45 kDa) protein expression. In immunofluorescence, analysis (bottom) demonstrates a reduction in the number of HCoV-229E-infected cells pretreated with 1 or 10 ng/mL IFN-α. (**b**) Immunoblot (top) of whole-cell lysates collected from A549 cells stimulated for 24 hours with 0, 1, or 10 ng/mL of IFN-α followed by infection with IAV virus (MOIs 2 and 10). The amount of IAV-NP protein (56 kDa) is drastically reduced as the expression of MxA protein is increased. The number of IAV-infected cells decreased in IFN-a pretreated cells, as demonstrated by immunofluorescence assay (bottom). Viral proteins were detected with rabbit α-229E-N or α-IAV-NP-specific antibodies, and MxA was detected using a monoclonal mouse α-MxA antibody, and GAPDH was used as a loading control. The histograms show relative expression of virus proteins in uninduced and induced cells, estimated by measuring the intensity of the bands in ImageJ. The experiment was repeated three times with similar results. Significance was determined by χ^2^-test (Table S1). The scale bar is 400 µm.

Next, we analyzed whether type I IFNs show any antiviral activity against HCoV-229E by pretreating A549 cells with different doses of IFN-α (1 or 10 ng/mL, 24 h) followed by an infection with HCoV-229E for 24 hours (MOI 10–0.04). IAV was used as a control. The expression of viral proteins and MxA in IFN-α pre-stimulated A549 cells was analyzed by immunoblotting and immunofluorescence (Fig. 5). IFN-α pretreatment inhibited IAV infection in a dose-dependent manner (Fig. 5b). The highest amount of IFN-α resulted in almost complete inhibition of IAV infection, with more than a 95% reduction in the number of virus-infected cells after IFN-α pretreatment compared to untreated cells. Both IAV infection and IFN-α pretreatment induced the expression of MxA in A549 cells, whereas HCoV-229E infection did not. Furthermore, IFN-α pretreatment led to a clearly detectable and significant (by χ^2^-test; Table S1) reduction in the number of HCoV-229E-infected cells (Fig. 5a), but the antiviral effect was much weaker compared with that observed against IAV infection. For example, the number of HCoV-229E-infected cells (MOI 0.4) was reduced by 40% and 80% in cells treated with 1 and 10 ng/mL IFN-α, respectively, compared to untreated cells (Table S1). Similar results were obtained by quantifying the amount of virus proteins produced following IFN-α pretreatment (that is, HCoV-229E N and IAV NP). Whole-cell lysates were analyzed by immunoblotting, and the intensity of viral protein-specific bands was assessed. In cells infected with MOI 2 of HCoV-229E, there was a roughly 30% and 50% reduction in N protein expression in cells pretreated with 1 and 10 ng/mL IFN-α, respectively, and with MOI 10, there was a 40% and 60% reduction in N protein expression, respectively. With IAV (MOI 2), there was a 60% and 80% reduction in NP expression levels for 1 and 10 ng/mL IFN-α, respectively, and for MOI 10, there was a roughly 70% and 95% reduction in NP for 1 and 10 ng/mL IFN-α, respectively. Taken together, these data indicate that while the replication of IAV is dramatically inhibited by pretreatment with IFN-α, HCoV-229E is somewhat resistant to the antiviral effects of type I IFNs.

### HCoV-229E infection in A549 cells almost completely blocks IFN-α-induced MxA protein expression

**Fig 6:**
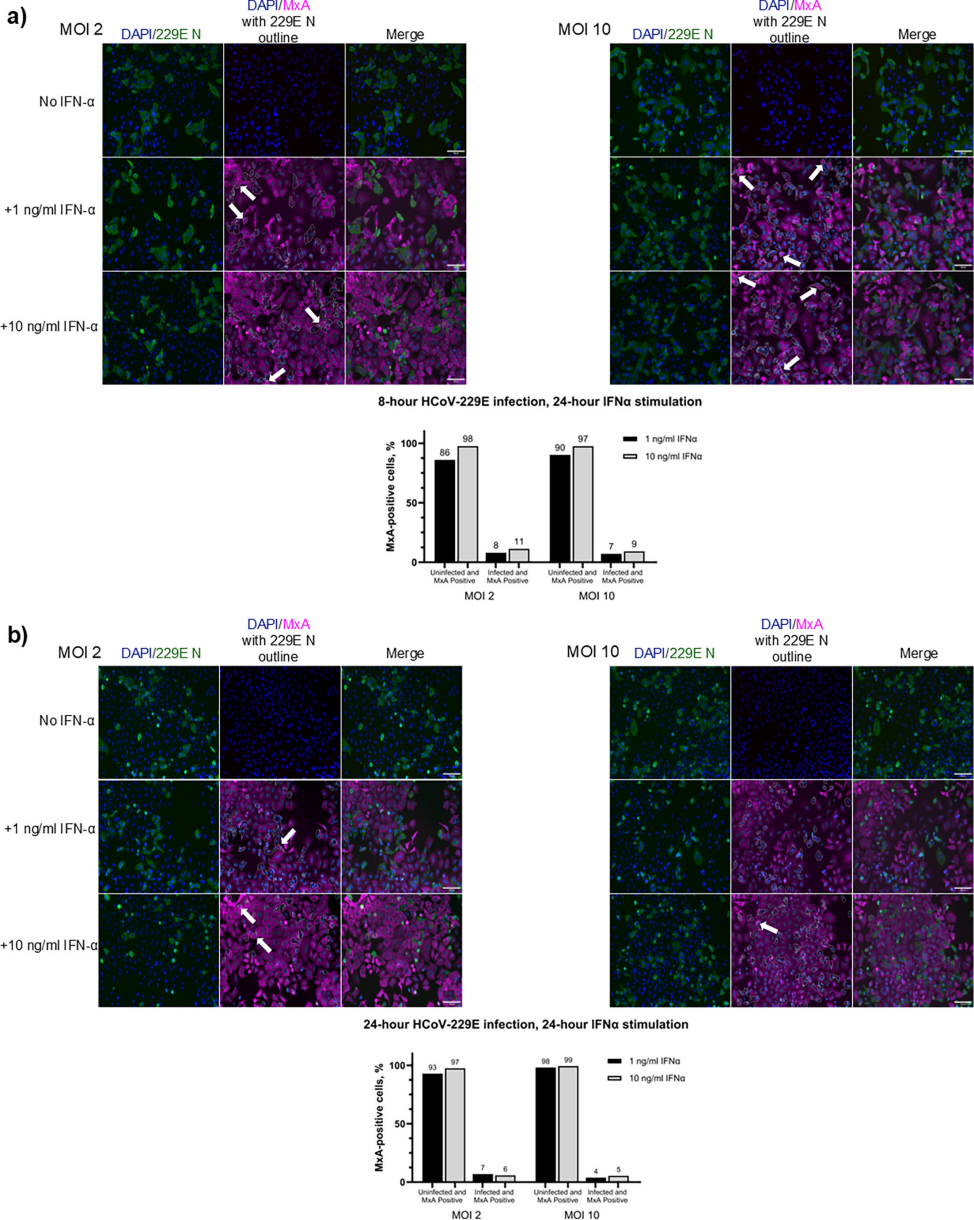
IFN-α-induced MxA protein expression is significantly inhibited in A549 cells infected with HCoV-229E. A549 cells were infected with HCoV-229E (MOI 2) for 8 or 24 hours before stimulation with IFN-α (1 ng/mL and 10 ng/mL) for 24 hours before fixing the cells and analyzing them with immunofluorescence microscopy. (**a**) Cells infected with HCoV-229E at MOI of 2 (left panel) or MOI of 10 (right panel) for 8 hours before the cells were stimulated with IFN-α followed by analysis of MxA expression. (**b**) Cells infected with MOI 2 (left panel) and MOI 10 (right panel) of HCoV-229E for 24 hours before IFN-α stimulation to assess MxA production. Virus infection was detected with rabbit α-229E-N antibodies (green), and MxA was detected with monoclonal mouse α-MxA antibodies (magenta). The histograms show the percentages of uninfected and infected cells, respectively, that are MxA-positive. White arrows indicate cells that are both virus- and MxA-positive. Altogether, the quantitation is based on the count of about 1,200–1,400 cells for the 8 hour infection and about 1,600–2,900 cells for the 24 hour infection, and significance was determined by χ^2^-test (Table S2). Experiments were repeated three times with similar results. Scale bar: 100 µm.

**Fig 7:**
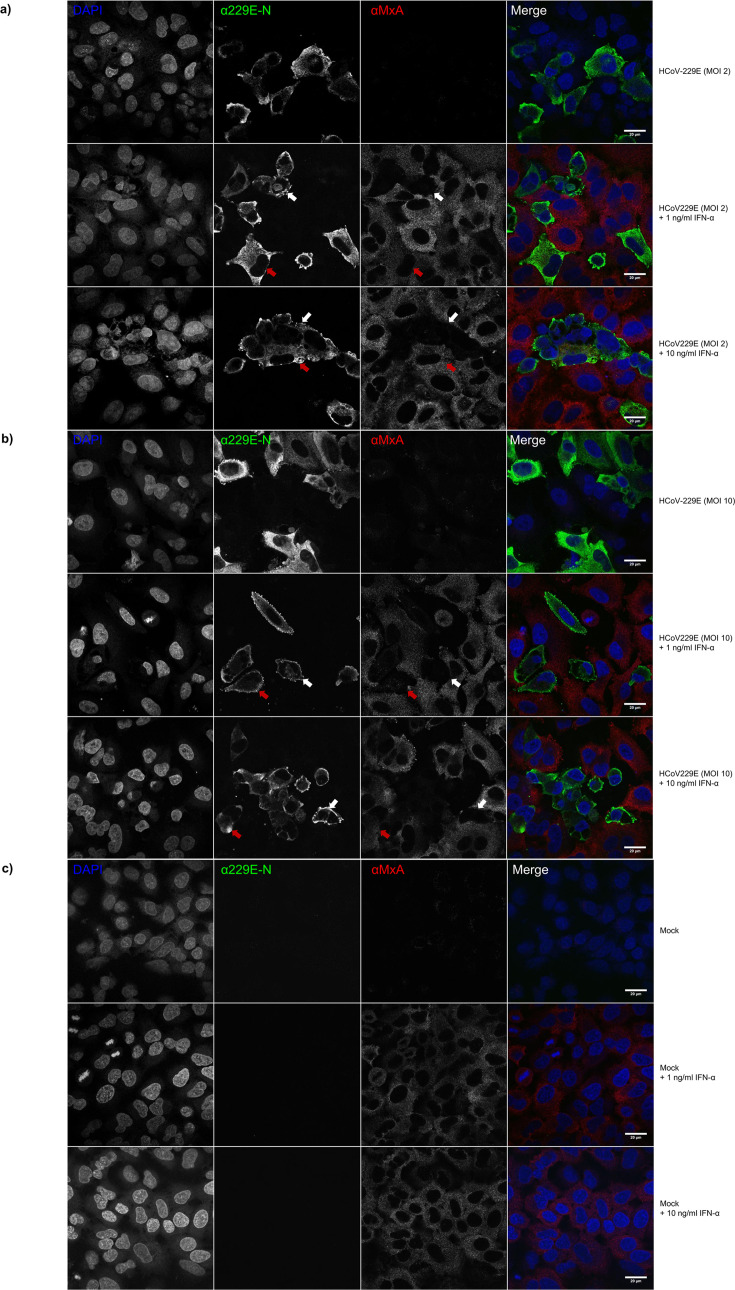
Confocal microscopic analyses of IFNα-induced MxA protein expression in 229E- and IAV-infected cells. To confirm the findings in Fig. 6, A549 cells infected with HCoV-229E for (**a**) 8 and (**b**) 24 hours before they were stimulated with IFN-α (1 ng/mL and 10 ng/mL) for 24 hours (mock cells in panel **c**). Cells were fixed, stained for both 229E N and MxA protein expression, and analyzed by confocal microscopy at 63× magnification (IAV images available in Fig. S4). Confocal images show 229E N protein-positive cells being mostly MxA-negative (white arrows), yet some cells that are both virus- and MxA-positive do exist (red arrow). Virus infection was detected with rabbit α-229E-N (green), and MxA was detected with monoclonal mouse α-MxA antibodies (red). Experiments were repeated three times with similar results. Scale bar: 20 µm.

We also analyzed whether HCoV-229E infection blocked type I IFN-induced gene expression. A549 cells were infected with HCoV-229E (MOI 2 and 10) for 8 or 24 h, followed by stimulation with IFN-α (1 ng/mL and 10 ng/mL) for 24 hours. IAV was used as a control. The expression of viral proteins and MxA was analyzed by immunofluorescence assay (Fig. 6 and 7). Infection with HCoV-229E MOI 2 and 10 showed similar infection rates, with roughly 25% of cells infected at 8 hpi MOI 2 and roughly 50% infected at 8 hpi with MOI 10. At 24 hpi, for both MOI 2 and 10, roughly 30%–35% of cells were infected (Fig. S6). The vast majority of A549 cells infected with HCoV-229E were devoid of MxA protein expression at 24 h after IFN-α stimulation (Fig. 6). However, some cells were double positive, that is, both virus- and MxA-positive, when induced with IFN-α. To further investigate and confirm this result, the samples were analyzed using high-resolution confocal microscopy (Fig. 7). The proportions of MxA-positive cells for both virus-positive and -negative cells were similar both at early (8 hpi, followed by 24 h stimulation with IFN-α) and later time points of the infection (24 hpi, followed by 24 h stimulation with IFN-α) (Fig. 6). Cells stimulated with IFN-α at 8 hpi showed positive MxA expression in more than 85% of uninfected cells, whereas only 10% of the infected cells were MxA-positive. The results were similar when infection doses MOI 2 or MOI 10 were used. When cells were stimulated with IFN-α at 24 hpi, at least 90% of uninfected cells and approximately 5% of the infected cells were MxA positive (significance was determined by χ^2^-test; Table S2). Figure 7 shows representative confocal microscope images of uninfected and HCoV-229E virus-infected cells followed by IFN-α stimulation and double staining for viral N and host MxA proteins. These data collectively indicate that HCoV-229E infection can efficiently inhibit IFN-α-induced MxA expression.

## DISCUSSION

In the present study, we demonstrate that the seasonal coronavirus HCoV-229E can replicate in certain human stable cell lines, such as A549 and Huh7 cells; however, no detectable IFN, other cytokines, or IFN-induced MxA gene expression was observed in response to viral infection. In addition, total cellular RNA from HCoV-229E-infected cells transfected into A549 lung epithelial cells did not stimulate IFN mRNA expression. IAV infection and cellular RNA from IAV-infected cells, instead, induced strong IFN-β and IFN-λ1 gene expression. Compared with IAV, HCoV-229E was less sensitive to the antiviral actions of type I IFNs (IFN-α2). Finally, we also observed that IFN-induced MxA protein expression was almost completely blocked in HCoV-229E-infected cells, indicating that type I IFN signaling was efficiently blocked by the infection.

Respiratory viruses have a profound impact on the immune system, influencing both the innate and adaptive immune responses. Upon infection, the genetic material of RNA viruses is detected by Toll-like receptors (TLRs) and/or RIG-I-like receptors (RLRs) depending on the virus or host cell type. Viral RNA recognition triggers signaling pathways that activate IFN (and other cytokine) production, followed by the activation of IFN-stimulated genes (ISGs) that mediate the antiviral actions of IFNs. Most, if not all, respiratory viruses have evolved strategies to evade or downregulate the host immune responses. These strategies include inhibiting PRR signaling, blocking IFN production and signaling, degrading or inactivating cytokines and IFNs, and modulating host gene expression to downregulate immune-related genes (29, 30).

The seasonal coronaviruses HCoV-229E, HCoV-OC43, HCoV-NL63, and HCoV-HKU1 are commonly associated with mild-to-moderate severity of respiratory illnesses, while SARS-CoV, MERS-CoV, and SARS-CoV-2 have been associated with moderate-to-severe infections in humans. Factors contributing to the pathogenesis of coronaviruses in humans may include the virulence of the virus, as well as the strength and quality of innate immune responses induced or downregulated by the viral infection. Surprisingly, in the present study, we observed that HCoV-229E infection in A549 and Huh7 cells did not induce any significant type I or type III IFN gene expression. In contrast to typical viral infections, in which the host innate immune signaling pathways promptly activate the production of IFNs, HCoV-229E seems to have evolved mechanisms to avoid or suppress the activation of IFN gene expression. Our data contradict those of some of the recent studies that demonstrated IFN production by seasonal CoVs (31), but this discrepancy may be due to the virus strains and experimental systems used in different studies. Conversely, a recent report (32) analyzed transcriptomic changes in MRC-5 cells, fibroblasts isolated from lung tissue, following infection with HCoV-229E, and found that there was significant downregulation of ISGs and other early antiviral response factors. Similar results were also reported by (33), where *in silico* transcriptomic analyses of MRC-5 cells infected with HCoV-229E revealed that when compared to more pathogenic coronaviruses such as SARS-CoV-2, HCoV-229E uniquely suppressed IFN signaling and failed to upregulate ISGs (33). Our data suggest that the HCoV-229E RNA is not recognized by the RIG-I receptors, and thus no activation of RIG-I, its adapter molecule MAVS, IKKε/TBK-1 kinases, IRF3 and NF-kB transcription factors, or type I and type III IFN gene expression takes place. This may indicate that the HCoV-229E vRNA molecules, RNA replication intermediates, or HCoV-229E-specific mRNA molecules structurally resemble those of the host cell RNA molecules. Thus, they are not recognized by PRRs, and the signaling cascades required for IFN production are not activated. While our data suggest HCoV-229E RNA evades RLR recognition, cell-type-specific IRF3/IRF7 activation may occur, as reported by Duncan et al. (34) In contrast, in our experiments IAV, whose genome consists of segmented negative-sense RNA molecules with a triphosphate structure at the 5′ end (strong RIG-I agonists), strongly activated IFN gene expression in A549 cells, indicating that our experimental system and approach can detect IFN gene expression with high sensitivity. This does not rule out the possibility that HCoV-229E, and perhaps other seasonal coronaviruses, encode proteins that can downregulate the RIG-I pathway and IFN gene expression. Such proteins and mechanisms have been described for IAV (NS1 [35–37]); and SARS-CoV-2 (Nsps [38]), the latter of which has been studied extensively during recent years. Further experiments are warranted to identify the structure of coronavirus-specific RNA molecules and potential viral IFN antagonists and their mechanisms of action for seasonal coronaviruses. Revealing the mechanisms of viral IFN antagonists may lead to new inventions that restrict the replication of this important group of viruses.

Another interesting observation was that type I IFNs showed antiviral activity against HCoV-229E; however, this activity was somewhat weaker than that observed against IAV. At present, it is not known which of the dozens of IFN-induced antiviral proteins are effective against HCoV-229E. One of the ISGs, the MxA protein, has been shown to be central to the antiviral response against several important RNA viruses, such as influenza viruses, Crimean-Congo hemorrhagic fever virus, and measles virus, but also against some DNA viruses, such as Mpox virus (35, 39–41). The mode by which Mx proteins elicit their inhibitory effects varies among viruses. For example, in the case of IAV infection, MxA interferes with the early stages of infection by preventing viral nucleocapsids from reaching the nucleus (15). As such, it is important to identify which of the antiviral proteins, such as PKR, OAS-RNase L, Viperin, Mx, or other ISGs mediate antiviral activity against coronaviruses. While MxA was our primary ISG marker, other ISGs, such as ISG15 or OAS1, should be investigated to fully elucidate HCoV-229E’s IFN evasion.

Additionally, since MxA production is induced by IFNs, specifically by type I or III IFNs (IFN-α/β or IFN-λ, respectively [11]), rather than directly by a virus, it is an excellent marker for IFN production and action (9). The differences in IFN and ISG induction and expression between the cell lines, where the observed lack of MxA in Huh7 cells reflects their weak IFN induction, unlike A549 cells, robustly express IFN and ISGs. We exploited this characteristic of the MxA gene and analyzed whether IFN-induced MxA protein expression was inhibited in HCoV-229E-infected cells. Interestingly, type I IFN-induced MxA expression was blocked in most cells at early time points (8 h) of HCoV-229E infection. The inhibition was even more dramatic at late stages of infection and was also evident when high IFN doses were used in the stimulation experiments. These data clearly show that type I IFN signaling is dramatically inhibited by HCoV-229E infection. It is known that MERS-CoV and SARS-CoV-2 encode multiple proteins that block IFN signaling at different stages of the signaling pathway (42). Future systematic analyses of whether seasonal coronavirus nonstructural, regulatory, or structural proteins can block IFN signaling may reveal interesting differences and similarities between different coronavirus family members. Identifying these differences and similarities may provide new common targets for the design of efficient antiviral substances against the entire coronavirus family.

In conclusion, unlike IAV, HCoV-229E does not induce early IFN expression in A549 or Huh7 cells, potentially due to RIG-I evasion or delayed activation, as seen in SARS-CoV-2 (27), and while seasonal coronaviruses have been shown to induce IFN production to some degree, their capacity to do so, at least in the case of HCoV-229E, is limited compared with that of more pathogenic coronaviruses. This restrained IFN production and response likely facilitates their ability to cause mild diseases while evading strong immune activation. Given the plethora of strategies employed by other viruses to inhibit and/or evade the early immune responses, it is likely that HCoV-229E may use a combination of these strategies. Since the HCoV-229E vRNA and viral mRNAs themselves do not seem to trigger an immediate innate immune response, this would allow HCoV-229E time to replicate and produce various proteins, such as Nsp1, which have been shown to inhibit innate immune signaling for several other coronaviruses. Taken together, this study suggests that potential strategies have been employed by HCoV-229E function, in part, to avoid the early activation of signaling pathways leading to IFN production along with downregulation of IFN signaling and antiviral protein expression. The relatively mild disease associated with HCoV-229E infection in humans may involve NF-κB or inflammasome pathways, which warrant further study to complement our *in vitro* findings. Ongoing research into the molecular mechanisms governing this interaction will provide further insights into the balance between viral evasion strategies and host immune defense mechanisms, with implications for understanding not only seasonal coronavirus infections but also coronavirus pathogenesis in general.

## SUPPLEMENTAL MATERIAL

Supplemental figures and tables spectrum.00510-25-s0001.pdf Figures S1 to S6 and Tables S1 and S2.

ASM does not own the copyrights to Supplemental Material that may be linked to, or accessed through, an article. The authors have granted ASM a non-exclusive, world-wide license to publish the Supplemental Material files. Please contact the corresponding author directly for reuse.
