# Supplementary material for: HCoV-229E does not trigger early interferon gene expression and evades IFN signaling in human A549 lung epithelial cells
Source: Microbiol Spectr. 2025 Oct 28;13(12):e00510-25. doi: 10.1128/spectrum.00510-25 (PMC12671147; doi:10.1128/spectrum.00510-25)
Supplement: Supplemental figures and tables — Figures S1 to S6 and Tables S1 and S2. [file spectrum.00510-25-s0001.pdf]

Suppl.Fig.1)

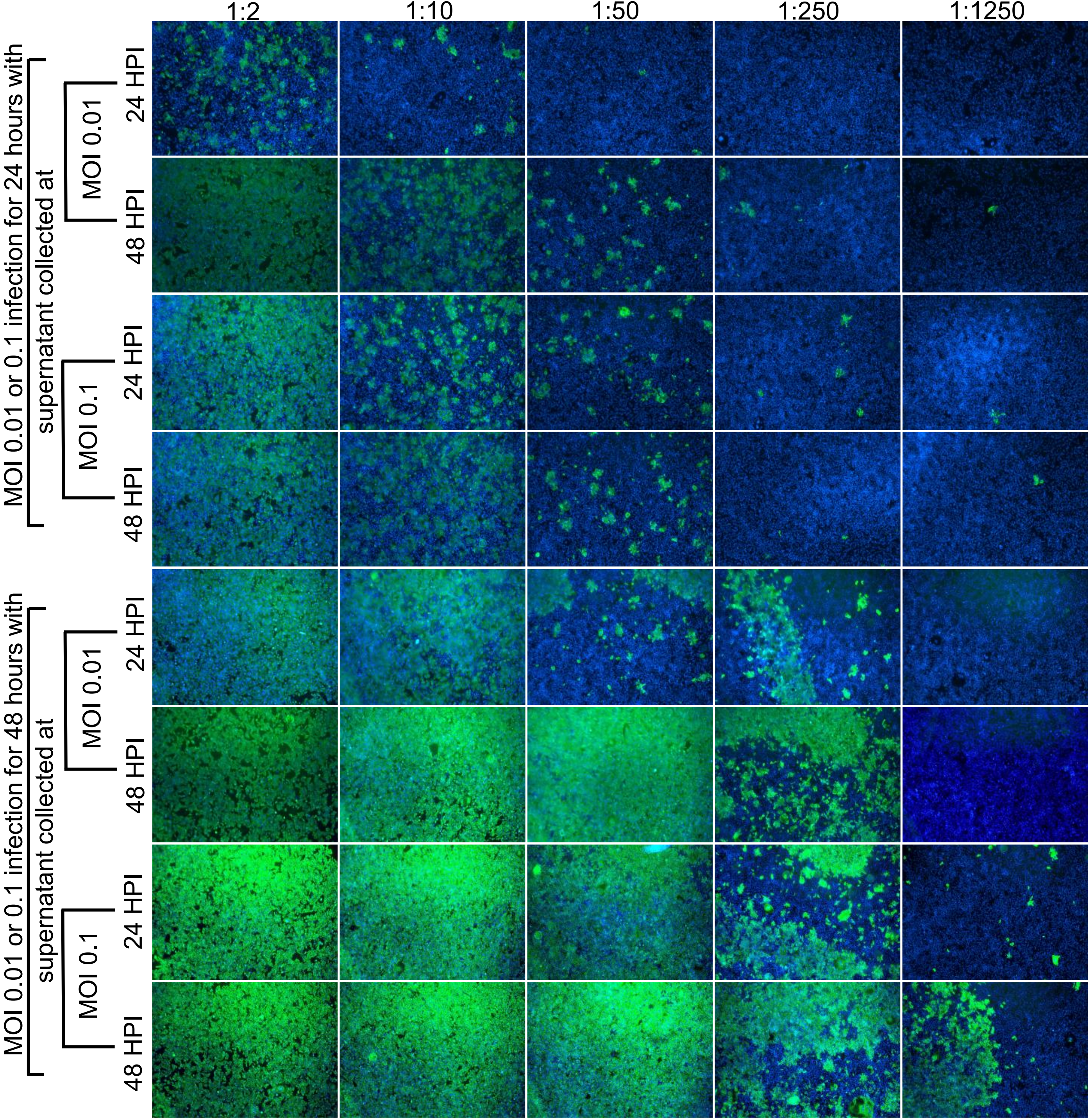
1:2
1:10
1:50
1:250
1:1250
MOI 0.01 or 0.1 infection for 24 hours with supernatant collected at
MOI 0.01
MOI 0.1
24 HPI
48 HPI
MOI 0.01 or 0.1 infection for 48 hours with supernatant collected at
MOI 0.01
MOI 0.1
24 HPI
48 HPI

Suppl.Fig.2)

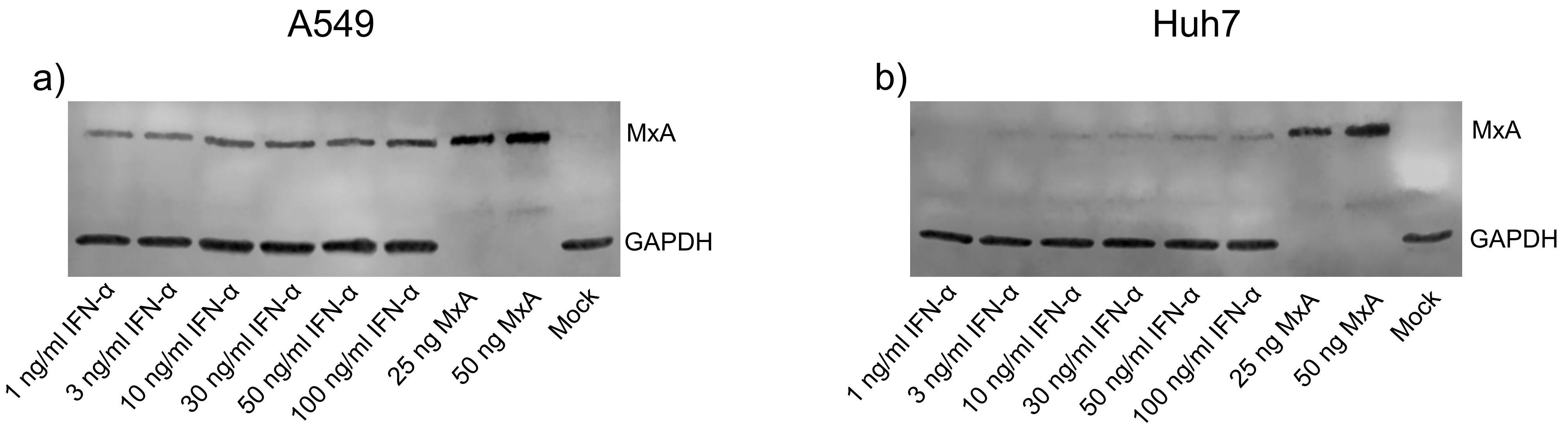
A549
a)
MxA
GAPDH
1 ng/ml IFN-α
3 ng/ml IFN-α
10 ng/ml IFN-α
30 ng/ml IFN-α
50 ng/ml IFN-α
100 ng/ml IFN-α
25 ng MxA
50 ng MxA
Mock
Huh7
b)
MxA
GAPDH
1 ng/ml IFN-α
3 ng/ml IFN-α
10 ng/ml IFN-α
30 ng/ml IFN-α
50 ng/ml IFN-α
100 ng/ml IFN-α
25 ng MxA
50 ng MxA
Mock

Suppl.Fig.3a)

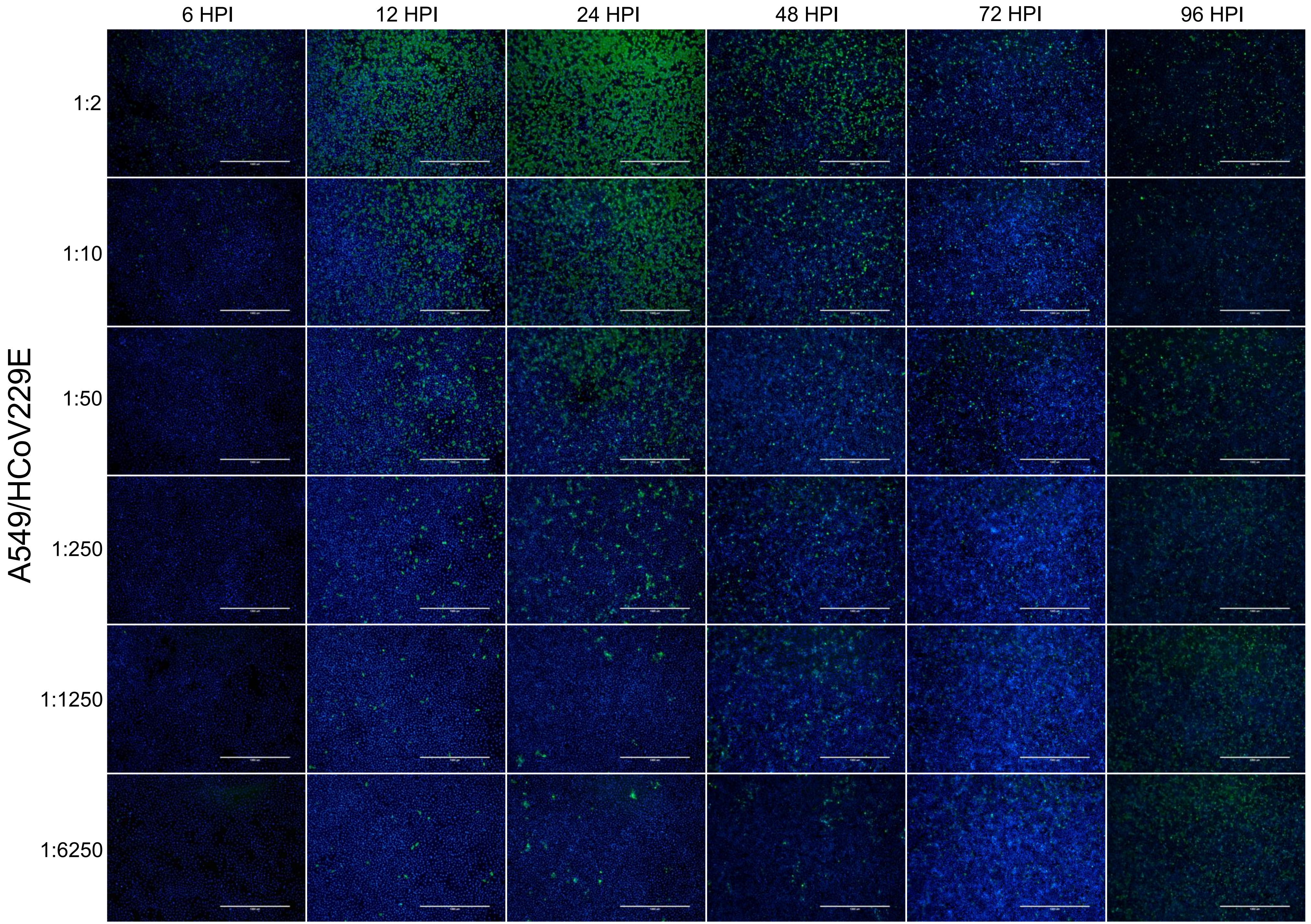

6 HPI
12 HPI
24 HPI
48 HPI
72 HPI
96 HPI
A549/HCoV229E
1:2
1:10
1:50
1:250
1:1250
1:6250

Suppl.Fig.3b)

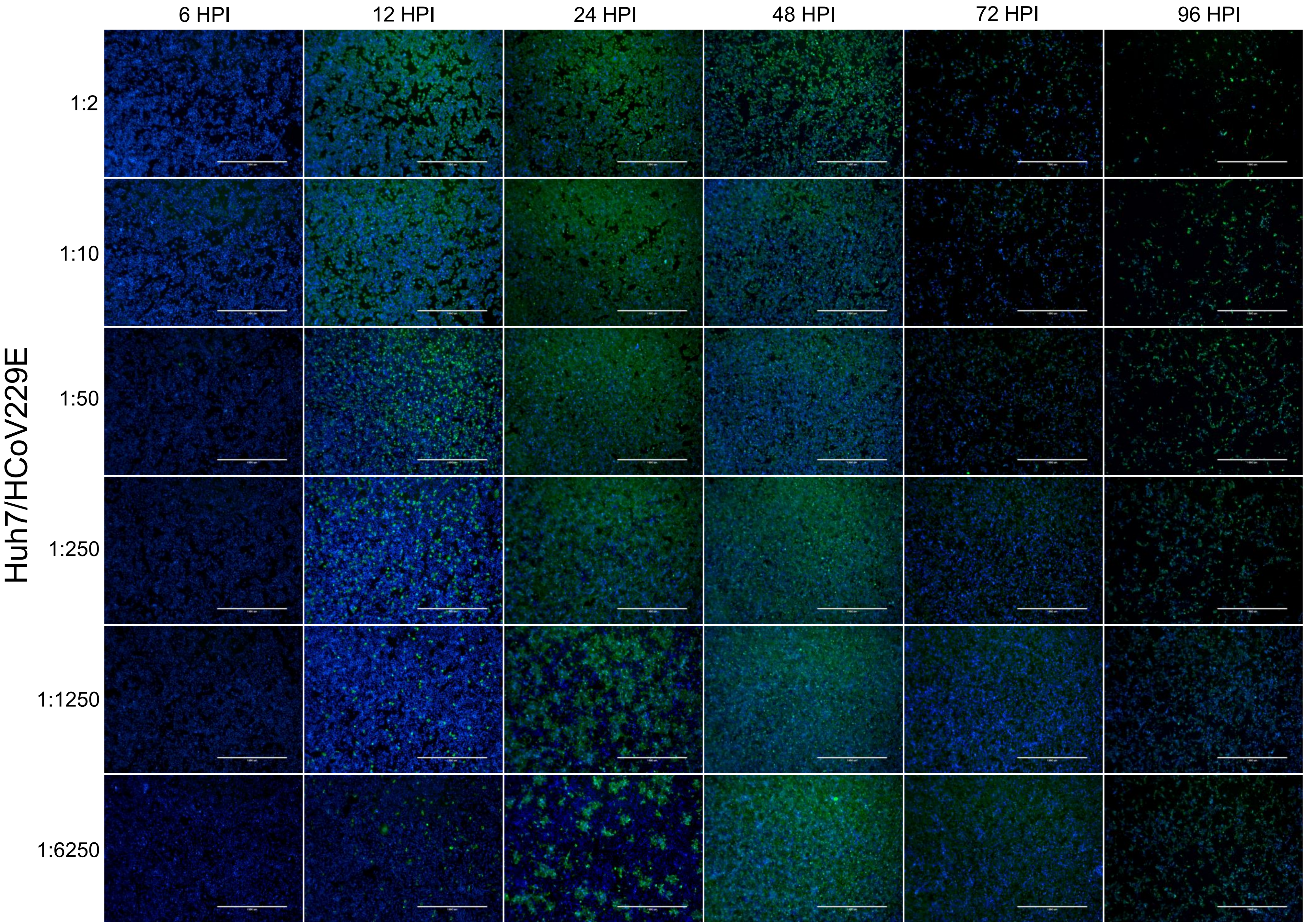
6 HPI
12 HPI
24 HPI
48 HPI
72 HPI
96 HPI
Huh7/HCoV229E
1:2
1:10
1:50
1:250
1:1250
1:6250

Suppl.Fig.3c)

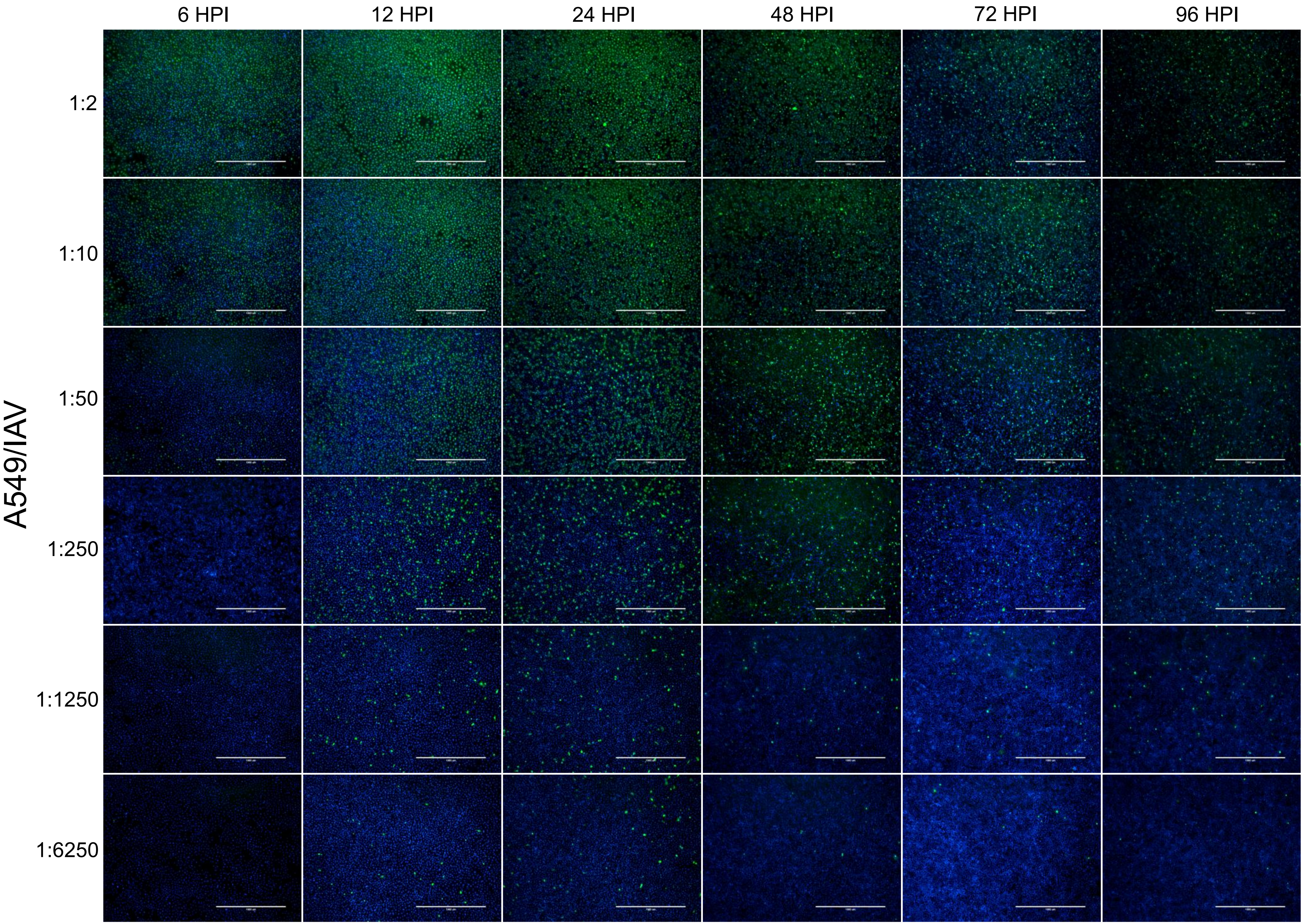
6 HPI
12 HPI
24 HPI
48 HPI
72 HPI
96 HPI
A549/IAV
1:2
1:10
1:50
1:250
1:1250
1:6250

Suppl.Fig.3d)

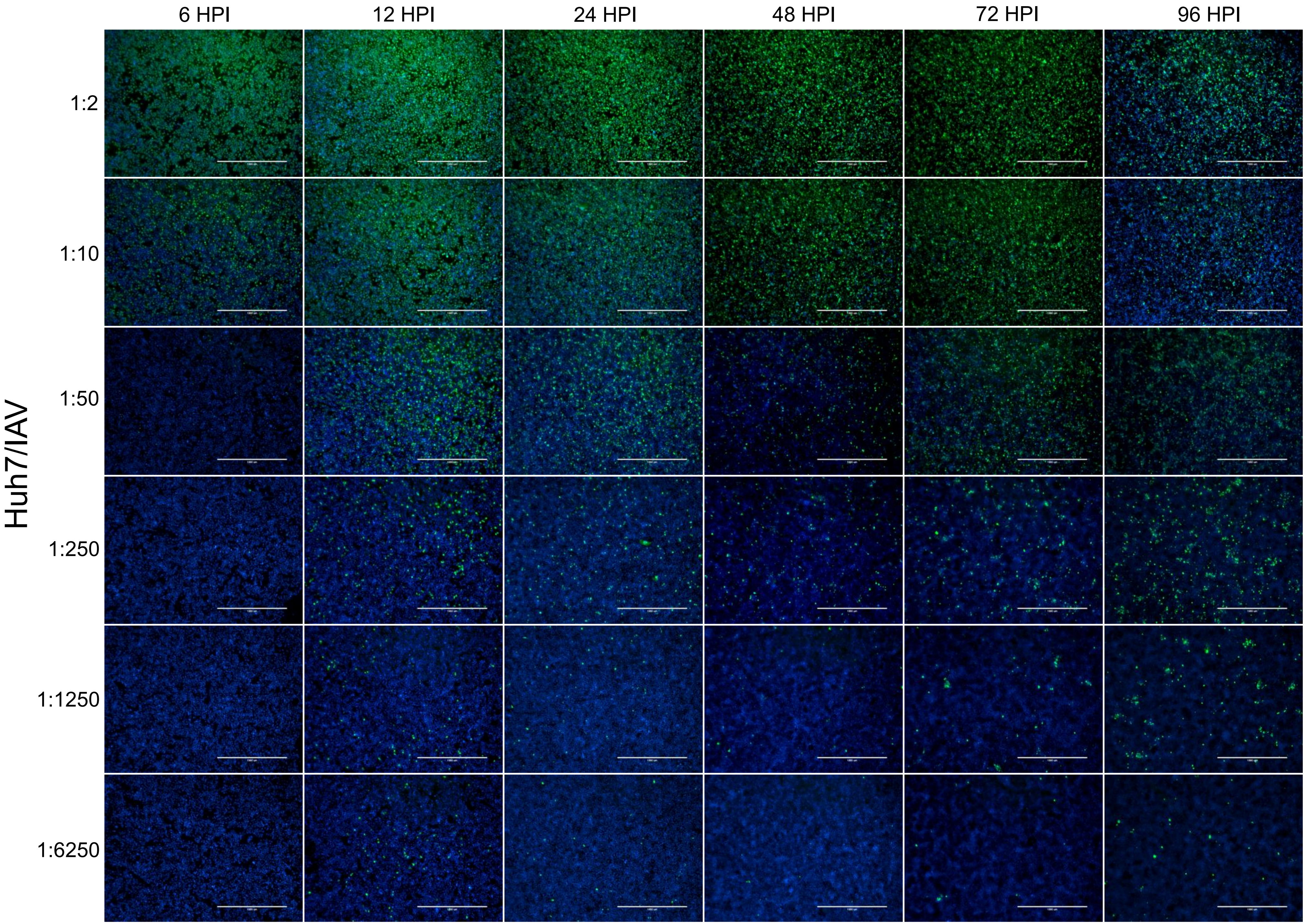

6 HPI
12 HPI
24 HPI
48 HPI
72 HPI
96 HPI
Huh7/IAV
1:2
1:10
1:50
1:250
1:1250
1:6250

Suppl.Fig.3d)

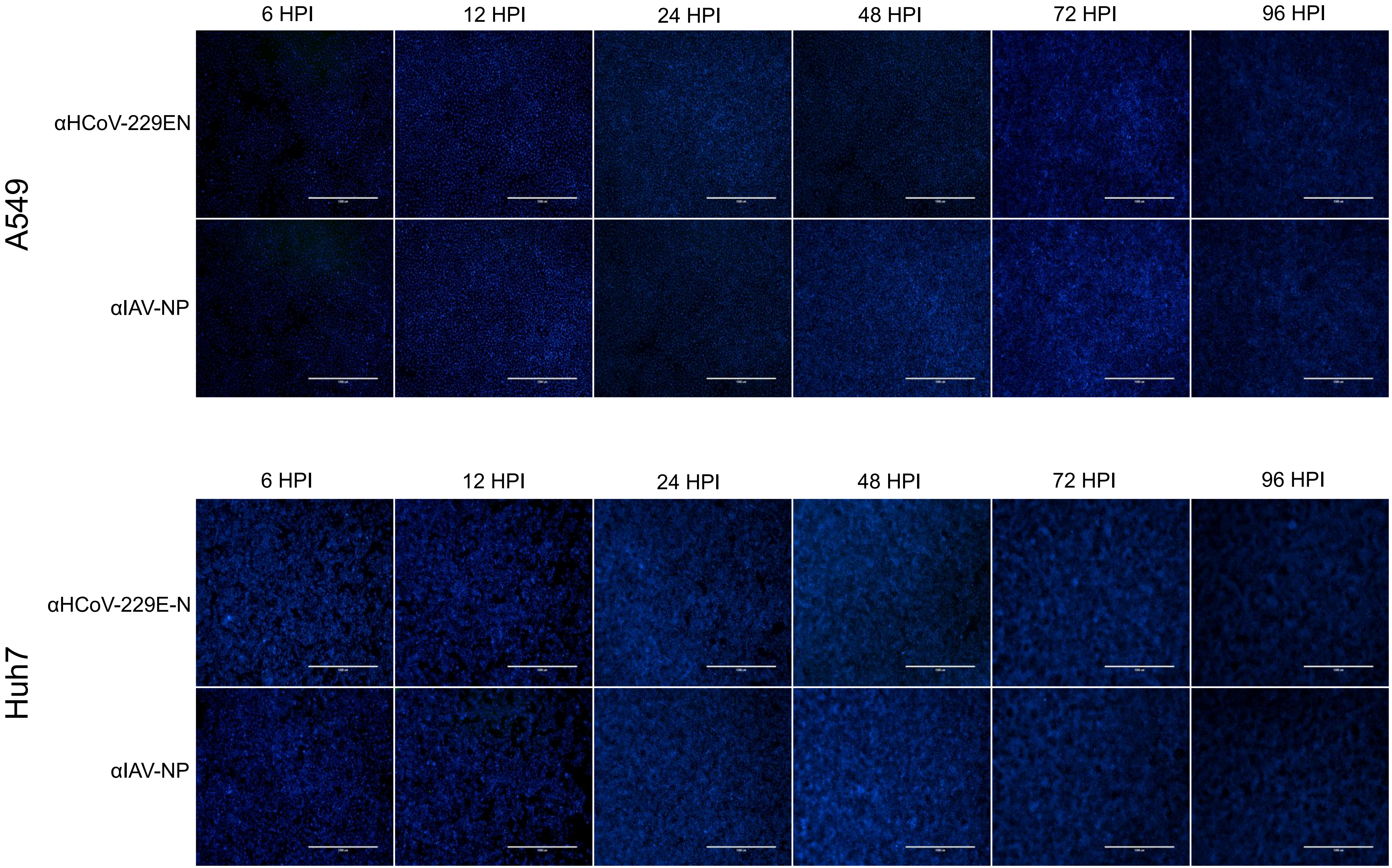
6 HPI
12 HPI
24 HPI
48 HPI
72 HPI
96 HPI
A549
αHCoV-229EN
αIAV-NP
6 HPI
12 HPI
24 HPI
48 HPI
72 HPI
96 HPI
Huh7
αHCoV-229E-N
αIAV-NP

Suppl.Fig.4

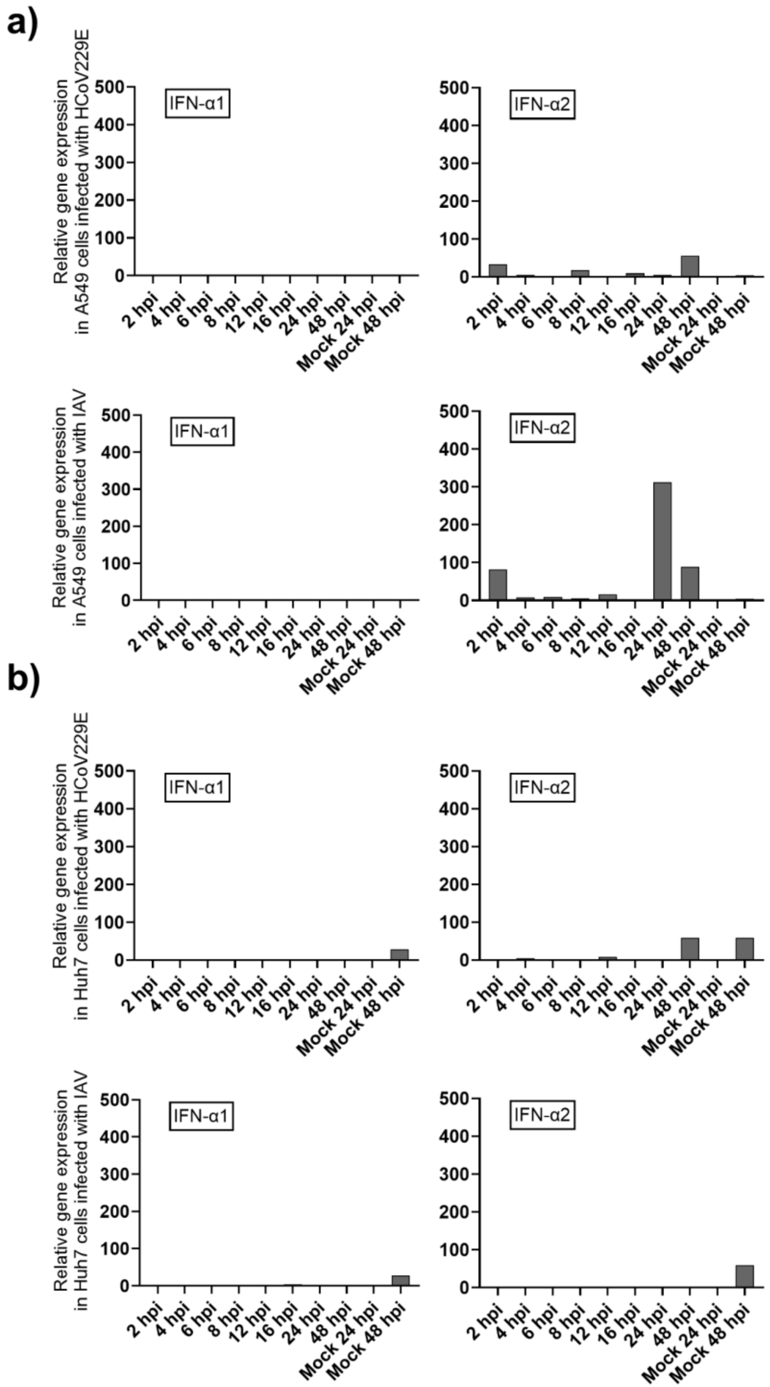
a)
Relative gene expression in A549 cells infected with HCoV229E
IFN-α1
IFN-α2
Relative gene expression in A549 cells infected with IAV
IFN-α1
IFN-α2
b)
Relative gene expression in Huh7 cells infected with HCoV229E
IFN-α1
IFN-α2
Relative gene expression in Huh7 cells infected with IAV
IFN-α1
IFN-α2
0
100
200
300
400
500
2 hpi
4 hpi
6 hpi
8 hpi
12 hpi
16 hpi
24 hpi
48 hpi
Mock 24 hpi
Mock 48 hpi

Suppl.Fig.5)

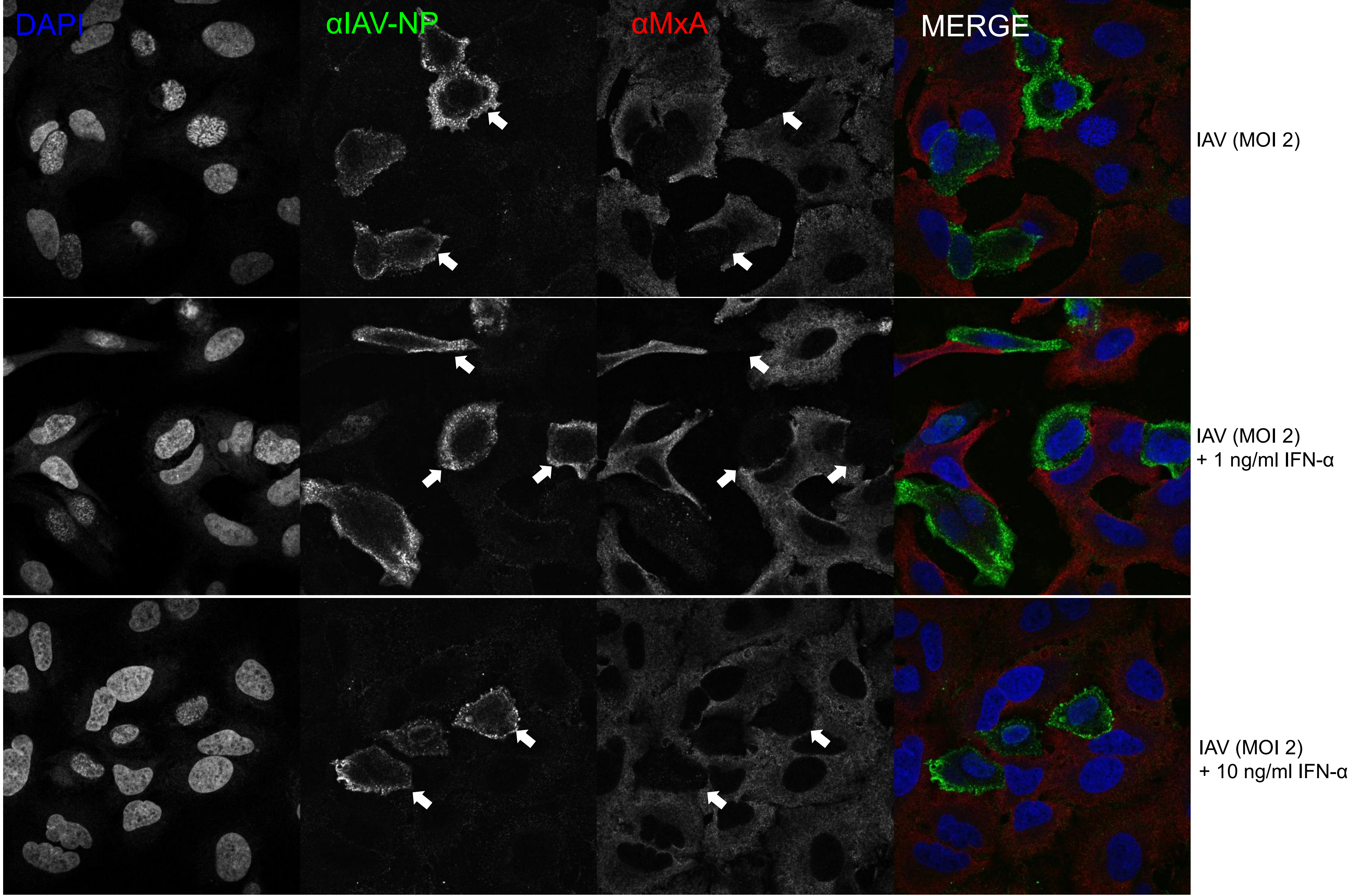
DAPI
αIAV-NP
αMxA
MERGE
IAV (MOI 2)
IAV (MOI 2)
+ 1 ng/ml IFN-α
IAV (MOI 2)
+ 10 ng/ml IFN-α

Suppl.Fig.5)

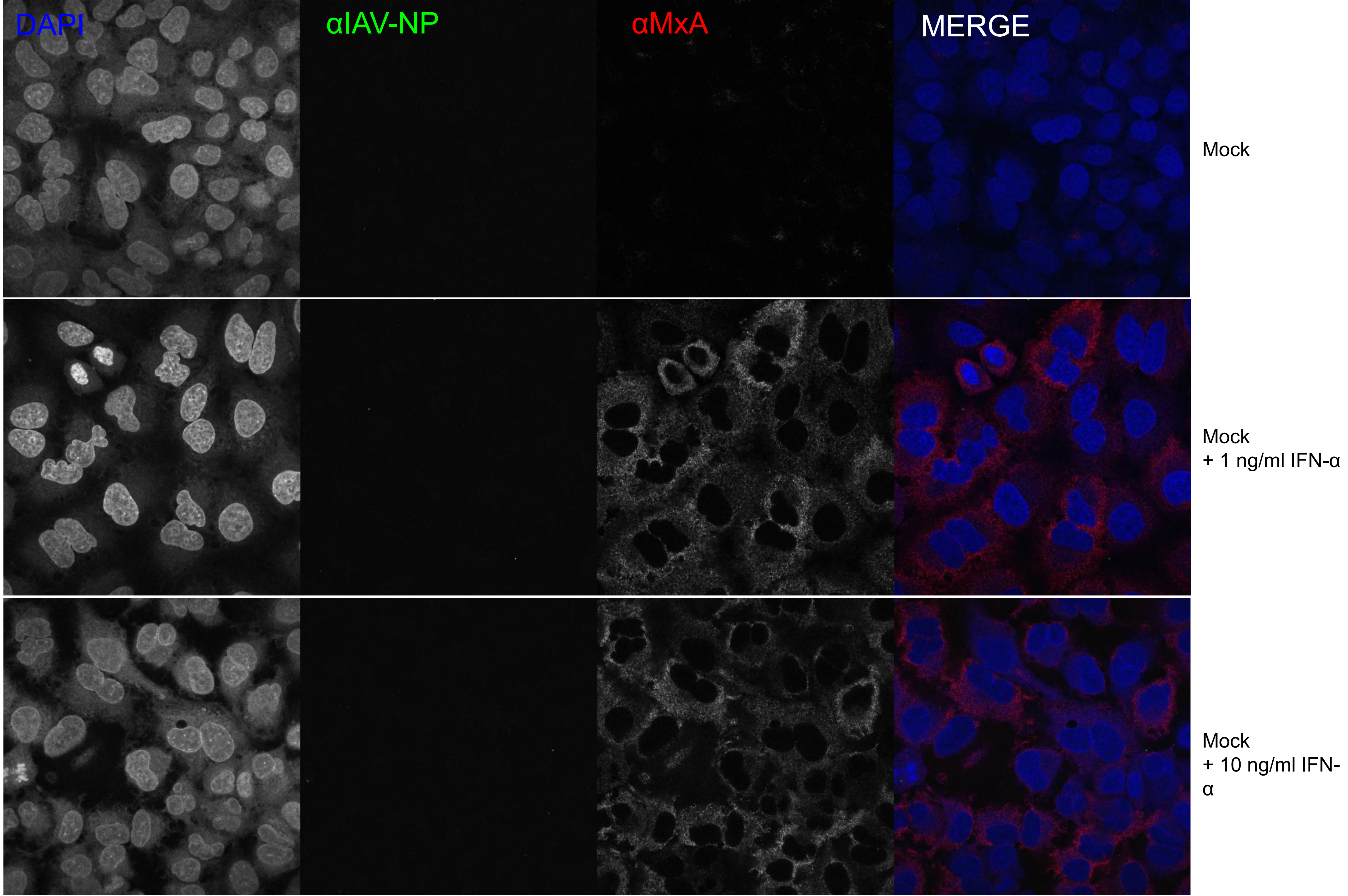
DAPI
αIAV-NP
αMxA
MERGE
Mock
Mock
+ 1 ng/ml IFN-α
Mock
+ 10 ng/ml IFN-
α

Suppl.Tbl.1

HCoV-229E MOI 2	Uninfected cells	Infected cells	Total cells
**No IFN-α**	253 (~51 %)	241 (~49 %)	494
**1 ng/ml IFN-α**	649 (~52 %)	598 (~48 %)	1247
**10 ng/ml IFN-α**	1058 (~82 %)	232 (~18 %)	1290

HCoV-229E MOI 0.4	Uninfected cells	Infected cells	Total cells
**No IFN-α**	1034 (~63 %)	607 (~37 %)	1641
**1 ng/ml IFN-α**	1730 (~76 %)	539 (~24 %)	2269
**10 ng/ml IFN-α**	2067 (~92 %)	195 (~8 %)	2262

Suppl.Tbl.2a

HCoV-229E MOI 2 8 hpi	Uninfected and MxA Positive	Uninfected and MxA Negative	Infected and MxA positive	Infected and MxA Negative
No IFN-α	0 (0 %)	886 (100%)	1 (1/315, 0.3 %)	314 (314/315, 99.7 %)
1 ng/ml IFN-α	819 (819/942, 86.9 %)	123 (123/942, 13.1 %)	26 (26/336, 7.7 %)	310 (310/336, 92.3 %)
10 ng/ml IFN-α	1064 (1064/1089, 97.7 %)	25 (25/1089, 2.3 %)	41 (41/414, 9.9 %)	373 (373/414, 90.1 %)

HCoV-229E MOI 10 8 hpi	Uninfected and MxA Positive	Uninfected and MxA Negative	Infected and MxA positive	Infected and MxA Negative
No IFN-α	0 (0 %)	636 (100 %)	1 (1/549, 0.2 %)	528 (528/549, 99.8 %)
1 ng/ml IFN-α	551 (551/612, 90 %)	61 (61/612, 10 %)	41 (41/668, 6.1 %)	617 (617/658, 93.9 %)
10 ng/ml IFN-α	725 (725/743, 97.6 %)	18 (18/743, 2.4 %)	62 (62/725, 8.6 %)	663 (663/725, 91.4 %)

Suppl.Tbl.2b

HCoV-229E MOI 2 24 hpi	Uninfected and MxA Positive	Uninfected and MxA Negative	Infected and MxA positive	Infected and MxA Negative
No IFN-α	0 (0 %)	1206 (100 %)	0 (0 %)	713 (100 %)
1 ng/ml IFN-α	1073 (1073/1156, 92.8 %)	83 (83/1156, 7.2 %)	25 (25/518, 4.8 %)	493 (493/518, 95.2 %)
10 ng/ml IFN-α	1104 (1104/1133, 97.4 %)	29 (29/1133, 2.6 %)	23 (23/428, 5.4%)	405 (405/428, 94.6 %)

HCoV-229E MOI 10 24 hpi	Uninfected and MxA Positive	Uninfected and MxA Negative	Infected and MxA positive	Infected and MxA Negative
No IFN-α	0 (0 %)	1871 (100 %)	1 (~0.1 %)	903 (~99.9 %)
1 ng/ml IFN-α	1843 (1843/1871, 98.5 %)	28 (28/1871, 1.5 %)	37 (73/1000, 3.7 %)	963 (963/1000, 96.3 %)
10 ng/ml IFN-α	2020 (2020/2035, 99.3 %)	15 (15/2035, 0.7 %)	47 (47/990, 4.7 %)	943 (943/990, 95.3 %)

Suppl.Fig.6a)

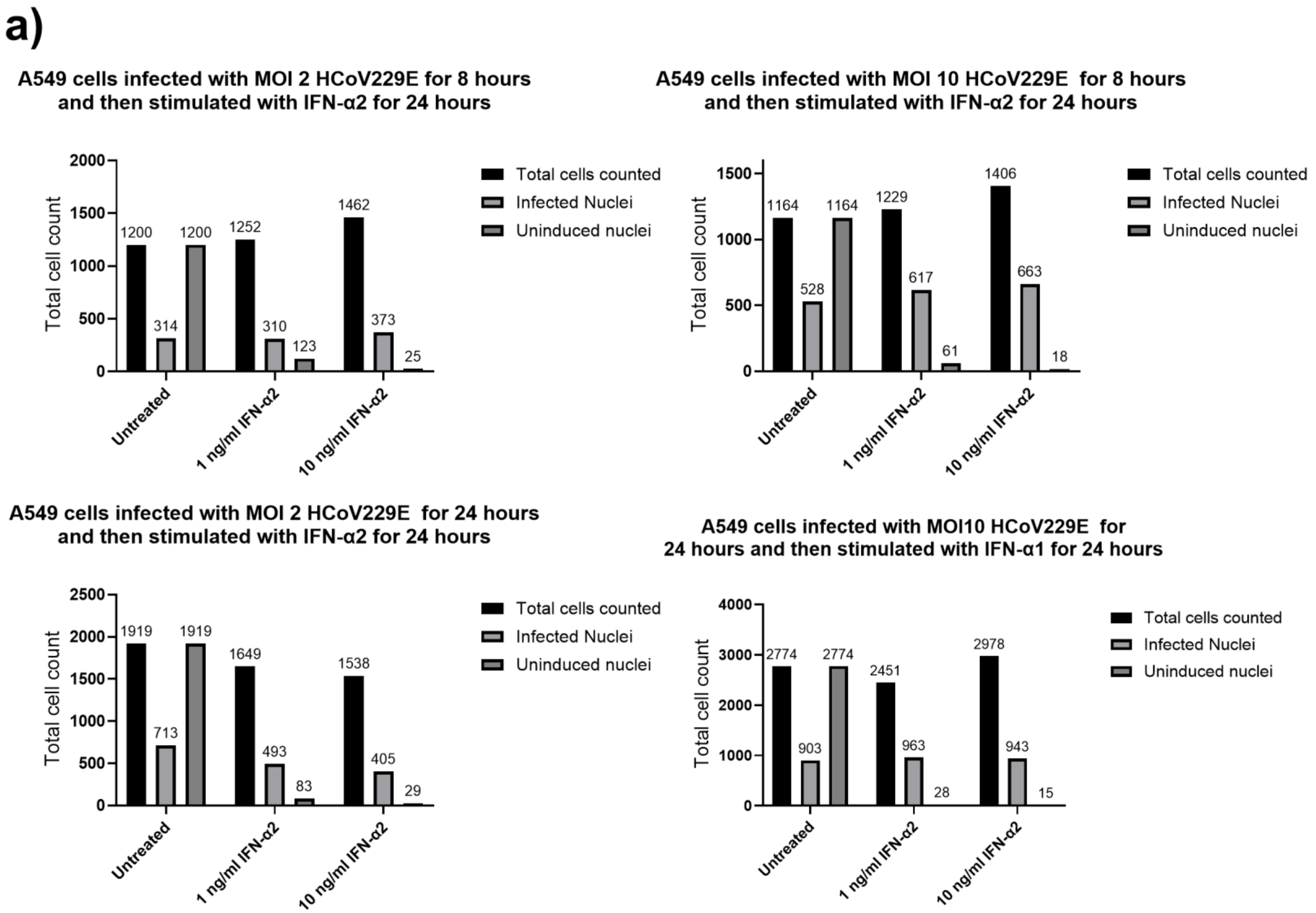
a)
A549 cells infected with MOI 2 HCoV229E for 8 hours and then stimulated with IFN-α2 for 24 hours
Total cell count
2000
1500
1000
500
0
1200
314
1200
1252
310
123
1462
373
25
Untreated
1 ng/ml IFN-α2
10 ng/ml IFN-α2
Total cells counted
Infected Nuclei
Uninduced nuclei
A549 cells infected with MOI 10 HCoV229E for 8 hours and then stimulated with IFN-α2 for 24 hours
1500
1000
500
0
1164
528
1164
1229
617
61
1406
663
18
A549 cells infected with MOI 2 HCoV229E for 24 hours and then stimulated with IFN-α2 for 24 hours
2500
2000
1500
1000
500
0
1919
713
1919
1649
493
83
1538
405
29
A549 cells infected with MOI10 HCoV229E for 24 hours and then stimulated with IFN-α1 for 24 hours
4000
3000
2000
1000
0
2774
903
2774
2451
963
28
2978
943
15

Suppl.Fig.6b)

b)

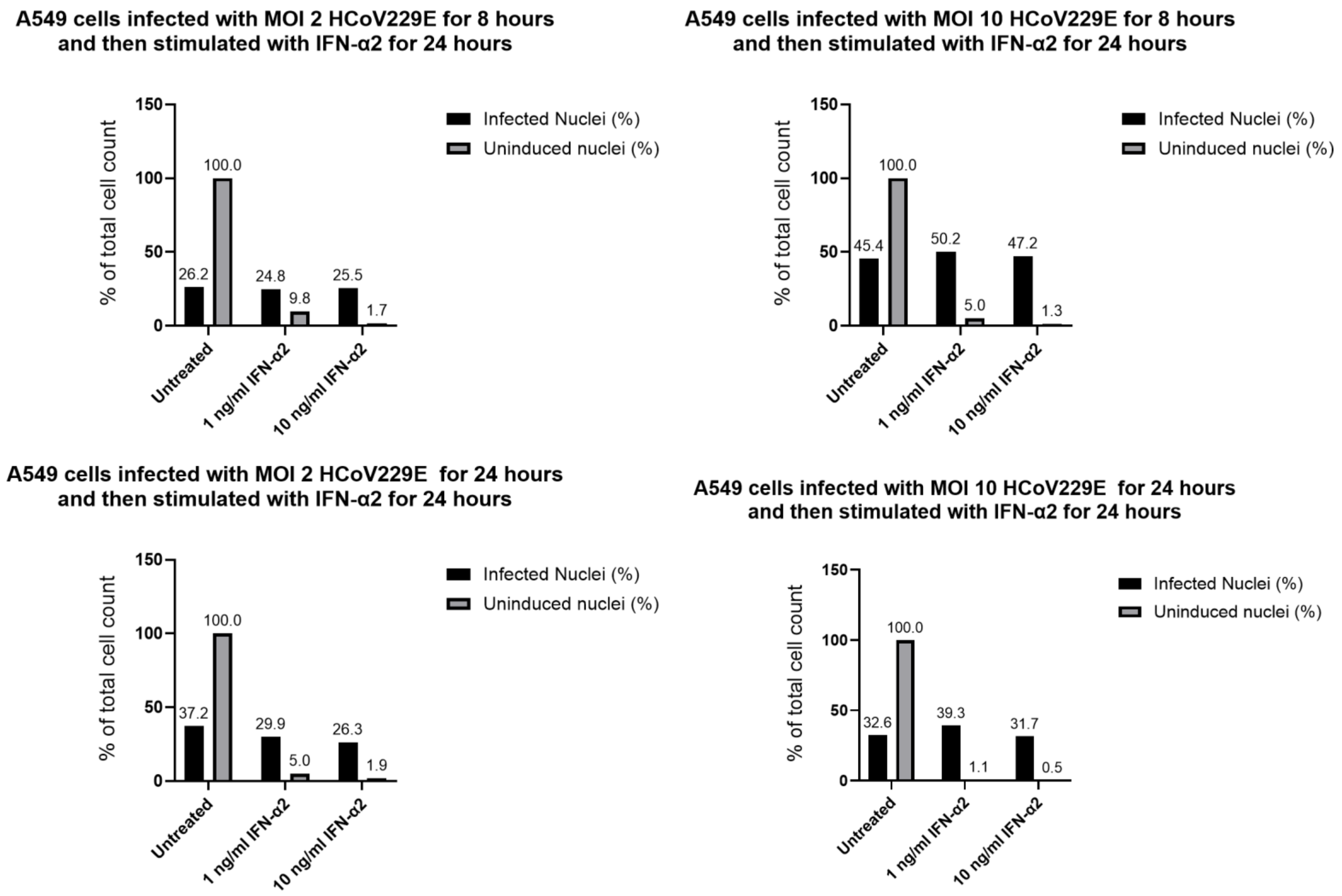

Suppl. Fig. 1) Reverse infectivity titer of different HCoV-229E p4 stocks. A HCoV-229E p3 stock was used to infect Huh7 cells with MOIs 0.01 and 0.1. Supernatant was collected at indicated time points and used to infect fresh Huh7 cells for 24 and 48 hours to assess infectivity of the p4 stocks. The cells were fixed in 4% formaldehyde at 24 hpi and then stained with αHCoV-229E-N. The MOI 0.1 48 hpi supernatant was considered the most infectious and used as a stock throughout the study.

Suppl. Fig. 2) IFN-α dose response in A549 and Huh7 cells. a) A549 cells and **b)** Huh7 cells were stimulated with the indicated dose of IFN-α for 24 hours. The cells were lysed and then analyzed on an immunoblot stained with α-MxA. As a positive control, 25 and 50 ng of pure MxA protein (76 kDa) was used and GAPDH (38 kDa) functioned as a loading control. In A549 cells, 1 ng/ml of IFN-α stimulation is sufficient for a clear signal and the maximum response is at 10 ng/ml IFNα stimulation. For Huh7 cells, 3 ng/ml IFN-α stimulation is required for a weak signal and the max response is seen with 30-50 ng/ml of IFN-α stimulation. Based on these results, 1 and 10 ng/ml IFN-α stimulation were used as induction controls in A549 cells and 3 and 30 ng/ml IFN-α stimulation were used as induction controls in Huh7 cells.

Suppl. Fig. 3) Infection kinetics of HCoV-229E and A/California/7/2009 in A549 and Huh7 cells. To assess the relative amounts required of each virus to reach 100% infection in 24 hours in each cell type, indicated dilutions of virus stock was added to Huh7 and A549 cells and the cells were fixed in 4% formaldehyde at indicated time points. **a)** HCoV-229E kinetics in A549 cells showing that 100% infection in A549 cells at 24 hpi was only possible with a 1:2 dilution of the p4 virus stock. **b)** HCoV-229E kinetics in Huh7 cells showing that 100% infection in Huh7 at 24 hpi cells was possible with a 1:250 dilution of the p4 virus stock. **c)** A/California/7/2009 kinetics in A549 cells showing that 100% infection in A549 at 24 hpi cells was possible with a 1:10 dilution of the virus stock. **d)** A/California/7/2009 kinetics in Huh7 cells showing that 100% infection in Huh7 at 24 hpi cells was possible with a 1:10 dilution of the virus stock. **e)** Mock A549 (top panel) and Huh7(bottom panel) cells stained at indicated time points with indicated antibodies. The conclusion of this kinetics experiment is that for HCoV-229E to reach 100% infection in 24 hours, a 1:2 dilution is required in A549 and a 1:250 dilution is sufficient in Huh7 cells and for influenza A, a 1:10 dilution of the stock is sufficient for both cell lines. The cells were stained with αHCoV-229E-N and αIAV-NP specific rabbit antibodies, respectively. The scalebar is 1000 μm.

Suppl. Fig. 4) IFN-α1 and -α2 expression in A549 and Huh7 cells following HCoV-229E or IAV infection. a) A549 cells and b) Huh7 cells were infected with HCoV-229E (top, MOI 10 for A549 and MOI 2 for Huh7) or IAV (bottom, MOI 10 for both A549 and Huh7) viruses. Cells were collected at indicated time points, total cellular RNA was isolated, and the expression of IFN-α1 and IFN-α2 mRNA was analyzed with RT-qPCR. All the relative values were calibrated using the 24 hpi mock sample value.

Suppl. Fig. 5) Confocal images of A/California/7/2009. A549 cells infected A/California/7/2009 virus (MOIs 2 and 10) for 8 and 24 hours before stimulation with IFN-α (1 ng/ml and 10 ng/ml) for 24 hours were investigated by confocal microscopy at 63x magnification. Confocal images show IAV-NP-positive cells being MxA negative (white arrows). Virus infection was detected with rabbit αIAV-NP antibodies (green), and MxA was detected with guinea pig α-MxA antibodies (red). Experiments were repeated three times with similar results. The scale bar is 20 μm.

Suppl. Tbl. 1) Tables of cells counted for significance tests for Fig 5. Analysis of antiviral effect of IFN-α against HCoV-229E infection. Immunofluorescence analysis demonstrates a reduction in the number of HCoV-229E infected cells pretreated with 0, 1, or 10 ng/ml of IFN-α for 24 hours before infection with MOI 2 (top) or MOI 0.4 (bottom). Cells were fixed at 24 h after infection and the number of uninfected and infected cells were counted in Fiji as described in Materials and Methods. χ^2-tests for independence were conducted to determine whether IFN-α pretreatment (0, 1, and 10 ng/ml IFN-α) is associated with reduced infection rate of cells (i.e., infected vs. uninfected). The tests were significant (For MOI 2: $\chi^2 = 295.99$, df = 2, $p < 0.0001$; For MOI 0.4: $\chi^2 = 458.74$, df = 2, $p < 0.0001$), indicating a strong association between IFN-α treatment and reduced infection rate.

Suppl. Tbl. 2) Total counts for infected and uninfected vs induced and uninduced cells for Figure 6. A549 cells were infected with HCoV-229E (MOIs 2 and 10) for **a)** 8 or **b)** 24 hours before stimulation with IFN-α (1 ng/ml and 10 ng/ml) for 24 hours before fixing the cells and analyzing them with immunofluorescence microscopy and cells were counted in Fiji as described in Materials and Methods. 1200-2900 cells were counted in total for each setting. χ^2-tests for independence were conducted to determine whether virus infection is associated with reduced MxA protein expression in IFN-α-stimulated (0, 1, and 10 ng/ml IFN-α) cells (i.e., infected vs. uninfected vs. induced vs. uninduced). For **a)**, in both cases $p < 0.0001$ (For MOI 2: $\chi^2 = 2586$, df = 6, $p < 0.0001$; For MOI 10: $\chi^2 = 1795$, df = 6, $p < 0.0001$) and for **b)**, in both cases $p < 0.0001$ (For MOI 2: $\chi^2 = 3029$, df = 6, $p < 0.0001$; For MOI 10: $\chi^2 = 5664$, df = 6, $p < 0.0001$) which indicates a statistically significant association between the conditions (IFN-α induced) and the distribution of MxA status (positive/negative) in both infected and uninfected cells.

Suppl. Fig. 6) Total cell, infected cell, and uninduced counts for 6. A549 cells were infected with HCoV-229E (MOIs 2 and 10) for 8 or 24 hours before stimulation with IFN-α2 (1 ng/ml and 10 ng/ml) for 24 hours before fixing the cells and analyzing them with immunofluorescence microscopy and cells were counted in Fiji as described in Materials and Methods. a) Total cell counts vs infected cells vs uninduced cells for untreated cells and cells treated with 1 or 10 ng/ml IFN-α2. b) Percentage of total cell counts for infected cells and uninduced cells for untreated cells and cells treated with 1 or 10 ng/ml IFN-α2.
